# Genome Comparison of Human and Non-Human Malaria Parasites Reveals Species Subset-Specific Genes Potentially Linked to Human Disease

**DOI:** 10.1371/journal.pcbi.1002320

**Published:** 2011-12-22

**Authors:** Christian Frech, Nansheng Chen

**Affiliations:** Department of Molecular Biology and Biochemistry, Simon Fraser University, Burnaby, British Columbia, Canada; The Centre for Research and Technology, Hellas, Greece

## Abstract

Genes underlying important phenotypic differences between *Plasmodium* species, the causative agents of malaria, are frequently found in only a subset of species and cluster at dynamically evolving subtelomeric regions of chromosomes. We hypothesized that chromosome-internal regions of *Plasmodium* genomes harbour additional species subset-specific genes that underlie differences in human pathogenicity, human-to-human transmissibility, and human virulence. We combined sequence similarity searches with synteny block analyses to identify species subset-specific genes in chromosome-internal regions of six published *Plasmodium* genomes, including *Plasmodium falciparum*, *Plasmodium vivax*, *Plasmodium knowlesi*, *Plasmodium yoelii*, *Plasmodium berghei*, and *Plasmodium chabaudi*. To improve comparative analysis, we first revised incorrectly annotated gene models using homology-based gene finders and examined putative subset-specific genes within syntenic contexts. Confirmed subset-specific genes were then analyzed for their role in biological pathways and examined for molecular functions using publicly available databases. We identified 16 genes that are well conserved in the three primate parasites but not found in rodent parasites, including three key enzymes of the thiamine (vitamin B1) biosynthesis pathway. Thirteen genes were found to be present in both human parasites but absent in the monkey parasite *P. knowlesi*, including genes specifically upregulated in sporozoites or gametocytes that could be linked to parasite transmission success between humans. Furthermore, we propose 15 chromosome-internal *P. falciparum*-specific genes as new candidate genes underlying increased human virulence and detected a currently uncharacterized cluster of *P. vivax*-specific genes on chromosome 6 likely involved in erythrocyte invasion. In conclusion, *Plasmodium* species harbour many chromosome-internal differences in the form of protein-coding genes, some of which are potentially linked to human disease and thus promising leads for future laboratory research.

## Author Summary

With more than 250 million infections and over a million deaths each year, malaria remains one of the most devastating infectious diseases worldwide. With the availability of complete genome sequences of both human and non-human *Plasmodium* parasites, the causative agents of malaria, it is now possible to use comparative genomics as a tool to look for genes that are present in some but not all *Plasmodium* species. Such species subset-specific genes possibly underlie important phenotypic differences between malaria parasites and could provide important clues for the development of new strategies to prevent and treat malaria in humans. In this study, we performed a comprehensive computational comparison of the published genomes of six *Plasmodium* species, including two human (*P. falciparum* and *P. vivax*), one monkey (*P. knowlesi*), and three rodent malaria parasites (*P. berghei*, *P. yoelii*, and *P. chabaudi*). This comparison revealed many species subset-specific genes that are potentially linked to human pathogenicity, human-to-human transmissibility, and human virulence. These genes can now be examined further by targeted experimental analyses to test predicted phenotypic associations and to elucidate gene function.

## Introduction

Malaria remains a serious health threat. Every year, more than 250 million people worldwide suffer from malaria and over one million people die as a consequence of the disease, mostly children in Africa under the age of five [1]. Malaria is an infectious disease caused by single-celled intracellular eukaryotic parasites of the genus *Plasmodium* that are transmitted by mosquitoes. Four species, *Plasmodium falciparum*, *Plasmodium vivax*, *Plasmodium malariae* and *Plasmodium ovale*, are traditionally recognized as human parasites. Other *Plasmodium* species are important model parasites in malaria research, including the primate malaria model *Plasmodium knowlesi*, which parasitizes macaque monkeys in the wild, as well as the three rodent malaria parasites *Plasmodium berghei*, *Plasmodium yoelii*, and *Plasmodium chabaudi*, which are natural parasites of thicket rats in central Africa.

*Plasmodium* species that naturally infect humans, monkeys, and rodents differ in their ability to cause human disease. Firstly, laboratory experiments have shown that parasites of thicket rats are infectious to various other species of rodents but not primates [2], [3], suggesting that rodent parasites lack essential features required to parasitize primates, including humans. Secondly, the macaque monkey parasite *P. knowlesi* differs from the four human parasites in that it is not endemic in larger parts of the human population despite its known ability to infect also humans under natural conditions [4]. Recent epidemiological and entomological data suggest that human *P. knowlesi* malaria is an ancient zoonosis acquired from forest-dwelling macaque monkeys [5]. It is likely that *P. knowlesi* malaria fails to spread in human settlements and beyond because of the known inability of *P. knowlesi* to develop in domestic species of *Anopheles* [6], [7]. However, concerns have been raised that with increased exposure of humans to *P. knowlesi* the parasite might eventually become epidemic in humans [4], [5]. Thirdly, human malaria parasites differ greatly in human virulence. *P. falciparum*, which accounts for up to 90% of annual infections worldwide [1], is the most virulent species and is responsible for almost all malarial deaths [8]. *P. vivax*, the major cause of human malaria outside Africa, rarely kills, although cases of lethal *P. vivax* malaria have been reported [9]. The more benign nature of *P. vivax* malaria in humans is commonly attributed to the inability of *P. vivax*-infected red blood cells to adhere to vascular endothelium and the preference of *P. vivax* to infect reticulocytes (immature red blood cells), which naturally limits parasitaemia because reticulocytes account for only 1–2% of erythrocytes [10], [11]. Finally, *P. vivax* and *P. ovale*, but not *P. falciparum* and *P. malariae*, can stay dormant in the liver as hypnozoites, which can cause relapses months or even years after the primary infection in the blood has been cleared [12]. Relapses are thought to be an evolutionary adaptation of the parasite to ensure transmission in more temperate climate zones where mosquitoes are not available throughout the year [11].

Recent genome sequencing of the two human malaria parasites *P. falciparum* [13] and *P. vivax* [14], the macaque parasite *P. knowlesi* [15], and the three rodent parasites *P. yoelii* [16], *P. berghei* [17], and *P. chabaudi* [17] provides an opportunity to identify the genetic basis of the aforementioned important phenotypic differences by means of comparative genomics. An important insight that has been gleaned from early comparative genomics analyses of *Plasmodium* genomes is that genes mediating parasite-host interactions are frequently restricted to a single *Plasmodium* species (species-specific) or restricted to a subset of *Plasmodium* species (species subset-specific). Perhaps the best studied and clinically most relevant example is *P. falciparum erythrocyte membrane protein 1* (PfEMP1), whose different isoforms are encoded by about 60 members of the *P. falciparum*-specific *var* gene family [13], [18]. PfEMP1 proteins are expressed at the surface of infected red blood cells (iRBC) where they mediate adhesion to both uninfected erythrocytes and host endothelial cells. This causes a great deal of the severe clinical pathologies of *P. falciparum* malaria. PfEMP1 is therefore considered the prime virulence factor of *P. falciparum* malaria. Other important species- or species subset-specific gene families have been linked to host immune evasion, including the *var* and *rif/stevor* gene families in *P. falciparum*, *vir* in *P. vivax*, *SICAvar* and *kir* in *P. knowlesi*, and the *cir/bir/yir* family in rodent malaria parasites (reviewed in [19]). Erythrocyte invasion is another critical molecular process at the parasite-host interface facilitated by species subset-specific gene family members, including duffy-binding like (DBL) and reticulocyte-binding-like (RBL) gene family members [14] as well as serine repeat antigens (SERA) and merozoite surface proteins (MSPs), some of which are now leading targets in vaccine development (reviewed in [20], [21]). Comparative genomic studies also have shown that species- or species subset-specific genes in *Plasmodium* genomes are preferentially located at dynamically evolving subtelomeric regions of chromosomes that are completely devoid of synteny [14], [16], [22], [23]. In contrast, non-subtelomeric or chromosome ‘core’ regions (referred to as *chromosome-internal regions* in the following) were found to be highly syntenic and to contain comparably few gene differences between species. Nevertheless, important species- and subset-specific genes have been described in chromosome-internal regions as well, including members of the aforementioned *var*, MSP, and SERA gene families in *P. falciparum* [13], [16], [23] as well as MSP and RAD genes in *P. vivax* [14], the latter of which has been associated with *P. vivax* selectivity for young erythrocytes and/or immune evasion [24]. The *P. knowlesi* genome is particularly rich in chromosome-internal species- and subset-specific genes, which have been identified as surface antigens of the *SICAvar* and *kir* gene families, respectively [15].

The fact that parasite genes mediating parasite-host interactions are frequently restricted to a single or a subset of *Plasmodium* species suggests that the search for species subset-specific genes is a promising strategy to identify new candidate genes underlying host-specific adaptations of *Plasmodium* species, in particular adaptations to human hosts and anthropophilic mosquito vectors. Identification and characterization of such genes may hold the key for important insights into molecular processes contributing to human disease. For example, parasite-encoded molecular factors that can explain why *P. falciparum*, *P. vivax*, and *P. knowlesi* but not rodent malaria parasites are infectious to humans are currently unknown. The identification of such pathogenicity factors could lead to new strategies to treat malaria in humans. Similarly, genes allowing *P. falciparum* and *P. vivax* but not *P. knowlesi* to complete their life cycle in anthropophilic mosquito vectors have not been identified, although an understanding of the genetic basis of this difference in human transmission success could help to prevent future host switches from monkey to human and pave the way for new transmission blocking strategies. Regarding human virulence it is likely that *P. falciparum* contains additional virulence genes that await functional characterization. The identification of new virulence genes would enhance our understanding of virulence mechanisms and could lead to new therapeutic interventions to treat severe malaria in humans. Finally, an understanding of the molecular mechanism underlying *P. vivax* hypnozoite formation is currently entirely missing but urgently needed, because hypnozoites cannot be killed by most available antimalarial drugs and complicate malaria eradication efforts [11].

We hypothesize that chromosome-internal regions of *Plasmodium* genomes harbour currently unappreciated species differences in the form of protein-coding genes that contribute to human pathogenicity, human-mosquito-human transmissibility, and human virulence. Other than subtelomeric regions, which contain mostly large and readily identifiable species-specific gene families [17], gene differences in chromosome-internal regions are currently largely unexplored [22], [23]. The goal of this study was therefore to systematically identify and characterize species subset-specific genes in chromosome-internal regions of *Plasmodium* genomes. Although not all subset-specific genes are expected to be functional due to stochastic processes of gene birth-and-death, a recent study in *Drosophila* has shown that a significant fraction of genes that differ between species do have important phenotypic effects [25]. We focused on four specific comparisons. First, to identify genes possibly linked to human pathogenicity, we determined genes well conserved in the genomes of *P. falciparum*, *P. vivax*, and *P. knowlesi* but absent in rodent malaria parasites, *P. chabaudi*, *P. berghei*, and *P. yoelii*. Second, we identified genes possibly crucial for parasite transmission success between humans by looking for genes present in *P. vivax* and *P. falciparum* but absent in the macaque monkey parasite *P. knowlesi*. Third, we identified genes that possibly contribute to severe human malaria by looking for *P. falciparum*-specific genes comparing *P. falciparum* with its less virulent relative *P. vivax*. Finally, we identified genes that potentially define unique features of *P. vivax* malaria by looking for genes present in *P. vivax* but absent in other sequenced *Plasmodium* genomes. Each of these comparisons resulted in the identification of several species subset-specific genes, most of which with unknown function. We propose these genes as attractive starting points for follow-up experimental analyses to test predicted phenotypic associations and to further elucidate their functions.

## Results

### Gene model improvement in *P. vivax* and *P. knowlesi*

Six published *Plasmodium* genomes sequenced to high coverage (ranging from 4 to 14.5-fold coverage) were selected for comparison, including the two clinically most important human parasites (*P. falciparum* and *P. vivax*), one monkey parasite (*P. knowlesi*), and three rodent parasites (*P. chabaudi, P. berghei, and P. yoelii*) (Figure S1). Preliminary examination of *P. vivax* and *P. knowlesi* gene models (Text S1) indicated that many gene models in these two genomes are apparently missing or mispredicted. To facilitate comparative analysis, we therefore started our analysis by improving current *P. vivax* and *P. knowlesi* gene models with homology-based gene prediction programs, using validated *P. falciparum* gene models as queries (see Materials and Methods). In total, we identified 53 and 19 new protein-coding genes and revised 165 and 116 existing gene models in *P. vivax* and *P. knowlesi*, respectively, including 31 split or merged genes (Table S1 and Table S2). Figure S2 shows four typical examples of improved gene models, including a novel gene, a split gene that was merged, a merged gene that was split, and an elongated gene model.

### Rodent pathogens likely defective in thiamine (vitamin B1) biosynthesis

**Figure 1:**
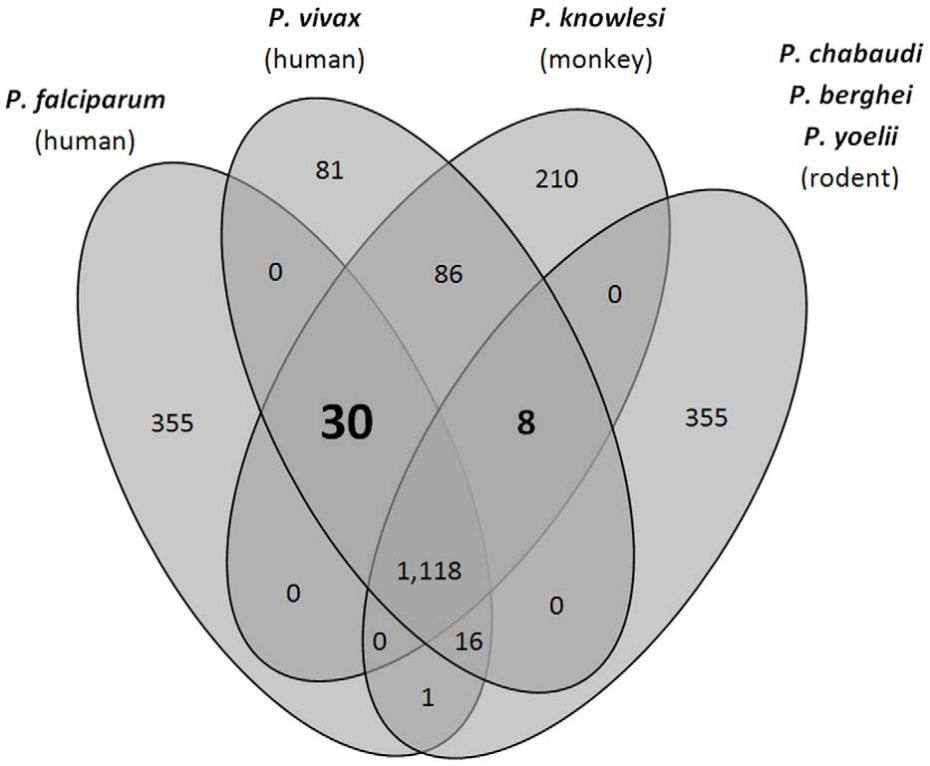
Proteome comparison reveals 30 proteins conserved in primate malaria parasites but absent in rodent malaria parasites. Genes were considered specific to a group of parasites if conserved in all in-group species (global protein sequence PID> = 40) but not in any of the out-group species (PID< = 15). In particular, primate-parasite specific genes are genes conserved in the three primate parasite proteomes but not in any of the three rodent parasite proteomes. The Venn diagram shows numbers of species subset-specific genes identified for all possible species combinations. Putative primate parasite-specific genes (30) are shown in bold. Note that gene numbers do not add up to species totals because genes with PIDs between 15% and 40% are not included.

Comparative analyses of the complete proteomes of *P. falciparum*, *P. yoelii*, *P. berghei* and *P. chabaudi*, and the improved proteomes of *P. vivax* and *P. knowlesi* using BLASTP and genBlastG (see Materials and Methods) identified 30 proteins well conserved in the three primate parasites (percent identity (PID) of global protein sequence alignment ≥40) that are putatively absent in all three rodent parasites (global PID≤15) (Figure 1). Examination of their identifiable syntenic genomic regions provided additional support for the absence of 16 of these putatively absent genes, *i.e.* no orthologous genes were found at the corresponding genomic positions in the rodent parasite genomes.

**Figure 2:**
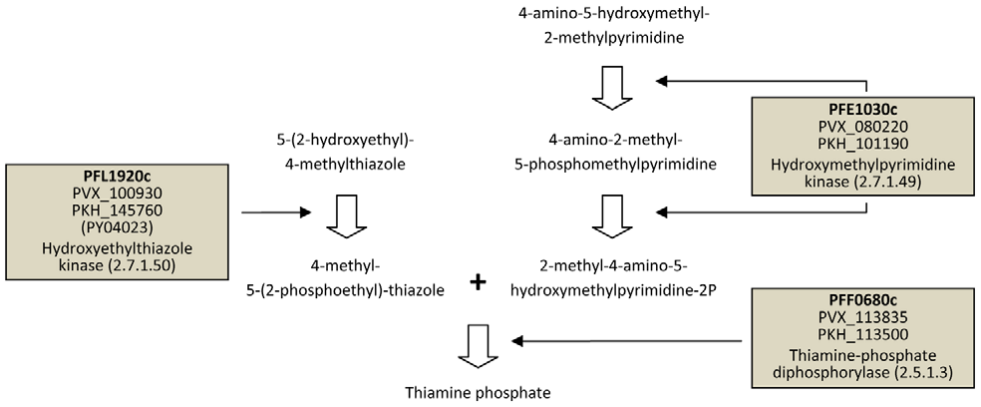
Rodent malaria parasites likely deficient in *de novo* synthesis of thiamine (vitamin B1). The diagram illustrates catalytic steps of the thiamine biosynthesis pathway in *P. falciparum*, with *4-amino-5-hydroxymethyl-2-methylpyrimidine* and *5-(2-hydroxyethyl)-4-methylthiazole* as start products and *thiamine phosphate* as the end product. The three enzymes predicted to be absent in rodent malaria parasites catalyze subsequent reactions in this pathway, suggesting that rodent malaria parasites are deficient in *de novo* synthesis of vitamin B1. Gene identifiers correspond to *P. falciparum* genes (bold) and their *P. vivax* and *P. knowlesi* orthologs (below). PFL1920c has a predicted but severely truncated ortholog in *P. yoelii* (PY04023). Figure based on pathway shown in the Malaria Parasite Metabolic Pathways (MPMP) database [26].

**Figure 3:**
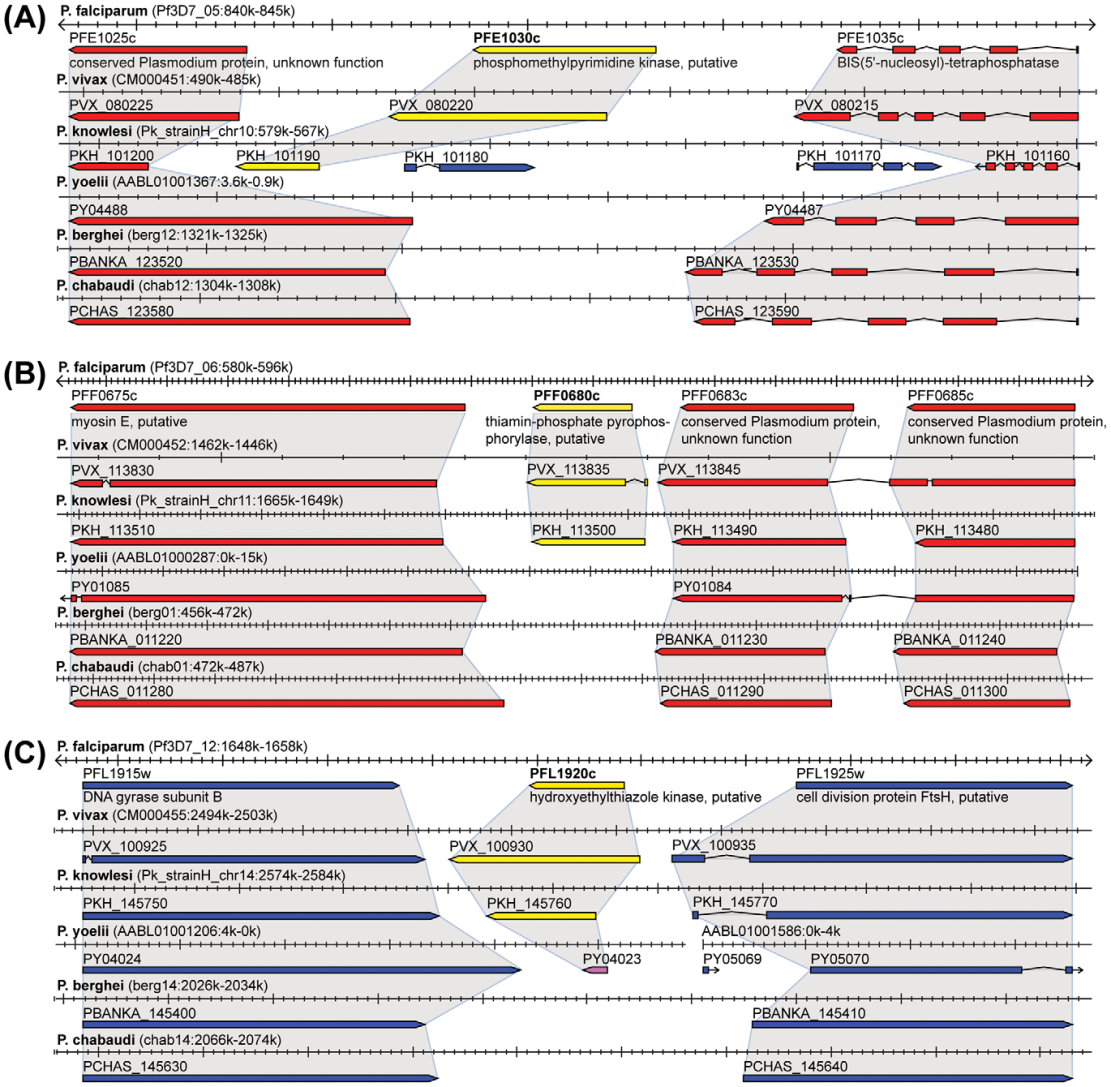
Syntenic orthologs of thiamine biosynthesis genes present in primate but not rodent parasites. Each panel shows one of the three *P. falciparum* thiamine biosynthesis gene on top (gene identifier in bold) and syntenic genomic regions in *P. vivax*, *P. knowlesi*, *P. yoelii*, *P. berghei*, and *P. chabaudi* below. Shaded areas indicate orthology. Thiamine biosynthesis genes displayed in yellow. Flanking genes and their orthologs on forward and reverse strand are shown in blue and red, respectively. The three *P. falciparum* genes are located on three different chromosomes and in all three cases syntenic orthologs are present in primate but not rodent parasite genomes. The syntenic *P. yoelii* ortholog of PFL1920c (PY04023, Panel C) is an exception but appears to be a truncated gene relic that should be annotated as pseudogene. Orthologs of PFF0683c and PFF0685c (Panel B) are merged into single genes in *P. vivax* and *P. yoelii* and should be split. Images adapted from PlasmoDB 8.0.

Table 1 shows *P. falciparum* orthologs of these 16 putative primate parasite-specific genes together with their degree of conservation in *P. vivax*, functional annotations, and expression profiles (see Table S9 for a list of the same genes but including graphical expression profiles). Among them are three key metabolic enzymes of the thiamine (vitamin B1) biosynthesis pathway: PFL1920c (hydroxyethylthiazole kinase, EC 2.7.1.50), PFE1030c (hydroxylmethylpyrimidine kinase, EC 2.7.1.49), and PFF0680c (thiamine-phosphate diphosphorylase, EC 2.5.1.3). Together, these three genes catalyze essential steps in the *de novo* synthesis of vitamin B1 (Figure 2). With one exception in *P. yoelii*, which appears to be a gene relic that should be annotated as a pseudogene, orthologs of all three *P. falciparum* genes are absent from three independent syntenic positions in all three sequenced rodent pathogen genomes (Figure 3). This data strongly suggests that primate but not rodent malaria parasites are capable of synthesizing vitamin B1 *de novo*.

**Table 1:** Genes conserved in primate parasites but absent in rodent parasites.

PfGene (PvPID/OG)	Product	RNA-seq expr. (IDC)	Protein expr.	GO biological process	Additional information
MAL13P1.214 (64/VIRI)	Phosphoethanolamine N-methyltransferase	Min: 0 hMax: 30 h	MZ;GC;SC;SZ;RU;TZ	Phosphatidylcholine biosynth. pr.	methionine and polyamine metabolism
MAL8P1.202 (53/ALVE)	apicoplast phosphate-idic acid phosphatase	Min: 15 hMax: 30 h	-	-	dolichol metabolism; SP
PF14_0565(44/----)	unknown function	Min: 10 hMax: 35 h	MZ	-	WD40 repeat-like
PF14_0036(69/ALVE)	acid phosphatase, putative	Min: 10 hMax: 25 h	MZ;GC;SC;TZ;RU	-	hydrolase activity
PF11_0186(65/----)	unknown function	Min: 10 hMax: 35 h	GC;SZ	-	-
PF14_0662(50/ALVE)	nucleoside transporter, putative	Min: 15 hMax: 30 h	-	-	nucleoside transmembrane transporter
PFF0680c(40/FIRM)	thiamin-phosphate pyrophosphorylase	Min: 15 hMax: 35 h	MZ	thiamine bio-synthetic process	-
PFE1030c(67/PROT)	phosphomethylpyrimidine kinase, putative	Min: 20 hMax: 35 h	SC;SZ;RU	thiamine bio-synthetic process	-
PFL1920c(76/FIRM)	hydroxyethylthiazole kinase, putative	Min: 15 hMax: 30 h	MZ;GC;TZ;SC;RU	thiamine biosynthetic process	-
MAL7P1.339 (46/ALVE)	Ca++ chelating serine protease, putative	Min: 20 hMax: 30 h	-	-	SCP/Tpx-1/Ag5/PR-1/Sc7 family; SP
PFI0405w(46/----)	unknown function	Min: 20 hMax: 35 h		-	1 TM; AP
MAL8P1.111 (42/ALVE)	JmjC domain containing protein	Min: 20 hMax: 35 h	GC	-	[Histone H3]-lysine-36 demethylase
PFL2255w(69/ALVE)	unknown function	Min: 25 hMax: 35 h	-	ubiquitin cycle	-
PFL1840w(66/ALVE)	unknown function	Min: 30 hMax: 40 h	GC;SZ	-	SP; 4 TM; COPI associated
PFL0305c(85/ALVE)	IMP-specific 5′-nucleotidase, putative	Min: -Max: -	GC; oocyst SZ	nucleotide metabolic process	magnesium ion binding; phosphatase activity
PFI1220w(47/ALVE)	unknown function	Min: -Max: -	-	-	acyl-CoA N-acyltransfer-ase; upregul. in GC/SZ

Genes conserved in *P. falciparum*, *P. vivax*, and *P. knowlesi* but absent in *P. berghei*, *P. chabaudi*, and *P. yoelii* as determined by genome-wide genBlastG searches and examination of syntenic genomic regions. *Min* and *Max* RNA-seq expression according to scaled expression values from the intraerythrocytic developmental cycle (IDC) as reported by [58]. A table with graphical RNA-seq expression profiles for these genes is provided in Table S9. Abbreviations: Pf: *P. falciparum*; Pv: *P. vivax*; PvPID: global protein sequence identity with *P. vivax* ortholog; OG: closest OrthoMCL DB species out-group with predicted ortholog of this gene; VIRI: Viridiplantae; ALVE: Alveolates; FIRM: Firmicutes; PROT: Proteobacteria; GO: gene ontology; SZ: sporozoites; (el)GC: (early/late) gametocytes; TZ: trophozoite; MZ: merozoites; SC: schizont; RU: rupture; AP: targeted to apicoplast; TM: predicted transmembrane domain; SP: predicted signal peptide.

Besides the three thiamine biosynthesis enzymes, Table 1 reveals additional enzyme-coding genes conserved in primate but absent in rodent malaria parasites. This includes an *acid phosphatase* (PF14_0036) involved in riboflavin (vitamin B2) metabolism [26], [27], a highly conserved putative *IMP-specific 5′-nucleotidase* (PFL0305c) that is involved in purine metabolism, an *apicoplast phosphatidic acid phosphatase* (MAL8P1.202) catalyzing the production of diacylglycerol as part of the dolichol metabolism [26], as well as two enzymes previously described as absent in rodent malaria parasites, including *phosphoethanolamine* N*-methyltransferase* (MAL13P1.214) that plays a role in phospholipid metabolism [28], and *Jumonji domain containing protein* (MAL8P1.111) serving as one of two functionally distinct *P. falciparum* histone lysine demethylases [29]. Other functionally annotated proteins conserved in primate malaria parasites but absent in rodent malaria parasites include a putative *nucleoside transporter* (PF14_0662), a putative *acyl-CoA* N*-acyltransferase* (PFI1220w) specifically upregulated in gametocytes and sporozoites [30], and a putative *Ca++ chelating serine protease* (MAL7P1.339). Taken together, the three primate parasites infectious to humans maintain a limited but conserved subset of genes that is absent in rodent malaria parasites, pointing towards new candidate pathogenicity genes required for parasitizing primate hosts, including humans.

### Synteny analysis reveals numerous gene differences in chromosome-internal regions

Further comparisons of the genomes of the three primate parasites *P. falciparum*, *P. vivax*, and *P. knowlesi* were performed using whole-genome synteny analysis. Synteny blocks were detected with OrthoCluster [31] based on our improved gene sets and orthology relationships predicted by Inparanoid (see Materials and Methods). Because our goal was to identify parasite-specific genes in syntenic chromosome-internal regions, we focused on the detection of *imperfect* synteny blocks (*i.e.* synteny blocks allowing for minor interruptions) and *non-nested* synteny blocks (*i.e.* synteny blocks not contained within larger synteny blocks due to one-to-many orthologous relationships) [32]. Note that in the context of pairwise synteny analysis we refer to genes without predicted ortholog in the other species as parasite-specific and not species-specific, because some of these genes might have predicted orthologs in other species.

**Figure 4:**
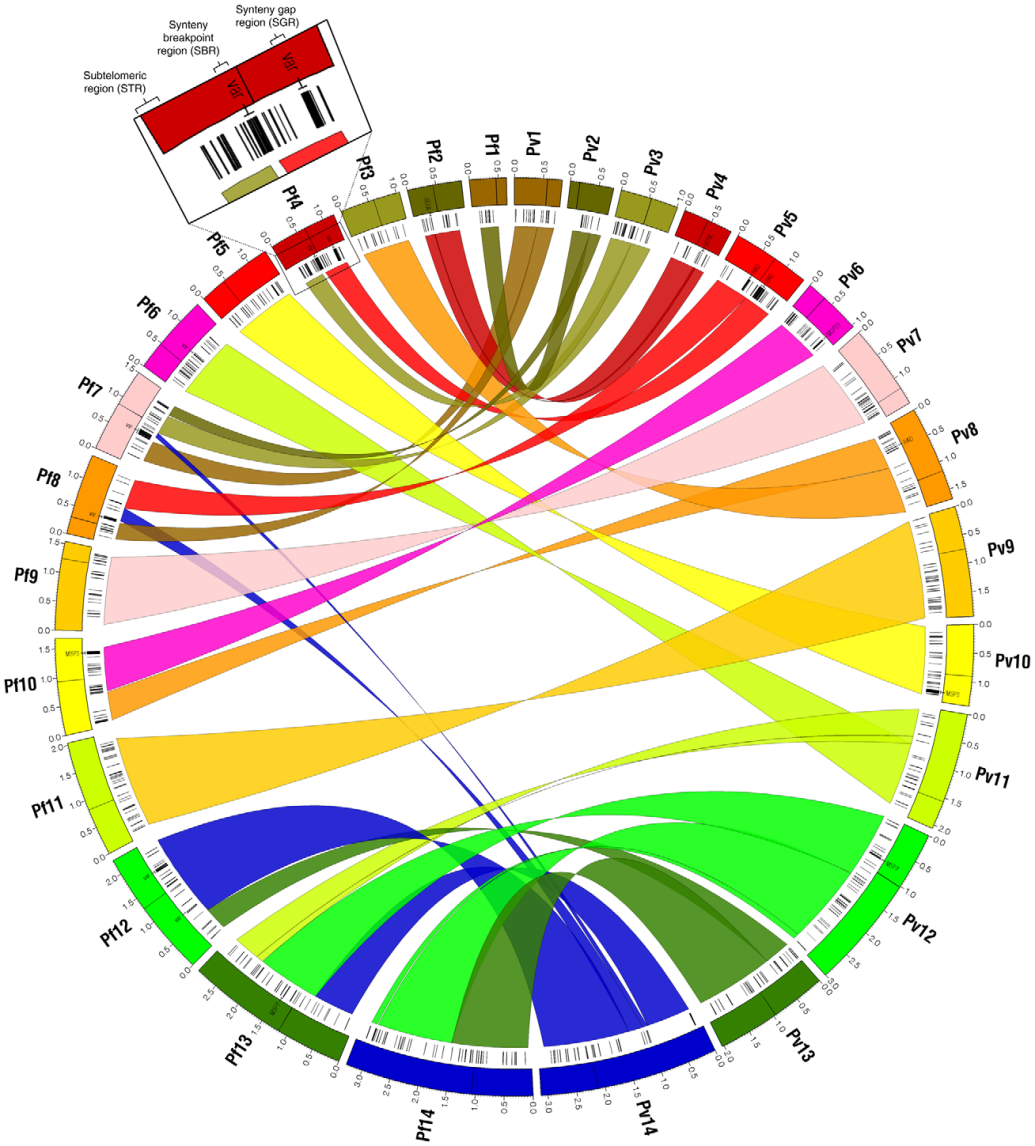
*P. falciparum* and *P. vivax* share extensive synteny with hundreds of putative parasite-specific genes in chromosome-internal regions. Outer segments depict the 14 nuclear chromosomes of *P. falciparum* (left semicircle, counter-clockwise) and *P. vivax* (right semicircle, clockwise). Each chromosome is assigned a different color. Ribbons indicate the 29 identified imperfect synteny blocks (28 non-nested and 1 nested) colored according to connected *P. vivax* chromosomes. Black tick marks underneath chromosomes indicate putative parasite-specific genes located at synteny gap regions (SGR) and synteny breakpoint regions (SBR) (see inset). Parasite-specific genes in subtelomeric regions (STRs) not shown. Text labels within chromosomes indicate parasite-specific genes mentioned in the text, including the newly identified putative MSP3 gene cluster on *P. vivax* chromosome 6. Black lines within chromosomes indicate putative centromeres. In both species, chromosome-internal regions contain hundreds of putative parasite-specific genes (388 in both species). Image created with Circos [56].

**Figure 5:**
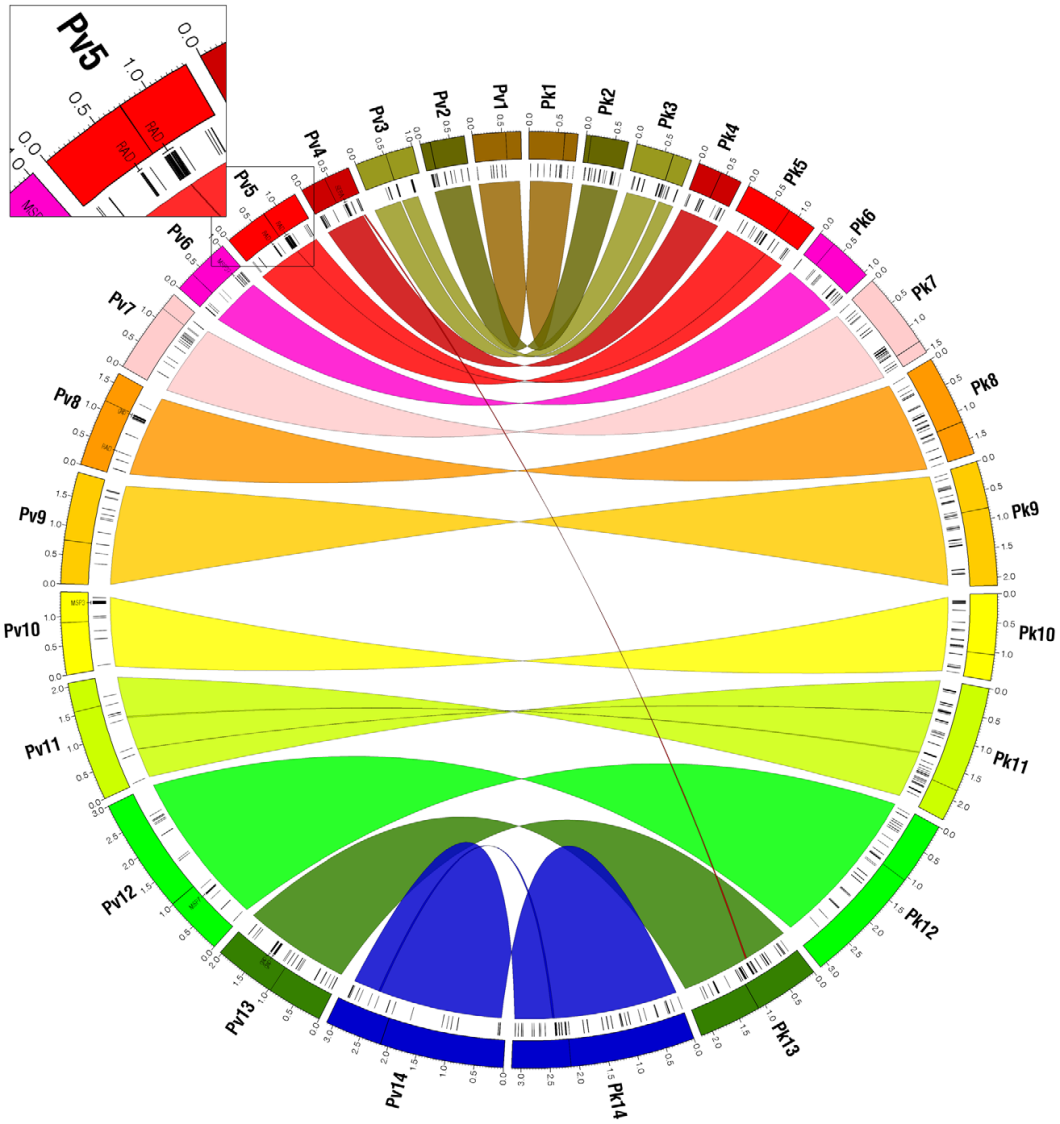
*P. vivax* and *P. knowlesi* share almost perfect 1-to-1 chromosomal synteny but also harbor hundreds of putative parasite-specific genes in chromosome-internal regions. Outer segments depict the 14 nuclear chromosomes of *P. vivax* (left semicircle, counter-clockwise) and *P. knowlesi* (right semicircle, clockwise). Ribbons represent the 20 identified imperfect synteny blocks (both nested and non-nested) colored according to connected *P. vivax* chromosomes. Black tick marks underneath chromosomes indicate putative *P. vivax*-specific genes (281) and *P. knowlesi*-specific genes (364) located at SGRs and SBRs. Parasite-specific genes in subtelomeric regions (STRs) not shown. Text labels within chromosomes indicate parasite-specific genes mentioned in the text. The inset shows the largest identified SGR in *P. vivax* containing 26 RAD genes. Black lines within chromosomes indicate putative centromeres. Excluding subtelomeres, imperfect synteny blocks span complete chromosomes with only two exceptions (*P. vivax* chromosomes 3 and 4), but also contain many putative parasite-specific genes, particularly in *P. knowlesi*. Image created with Circos [56].

Between the two human parasites *P. falciparum* and *P. vivax* (Figure 4 and Table S3), we identified 28 non-nested imperfect synteny blocks with a median size of 144.5 genes (563.7 kb) that collectively cover 90% of protein-coding genes or 85% of the nuclear genome sequence. Between *P. vivax* and *P. knowlesi* (Figure 5 and Table S3), OrthoCluster identified 16 non-nested imperfect synteny blocks of median size 300 genes or 1,376 kb (average of both genomes), each of them essentially spanning complete chromosomes with two exceptions on *P. vivax* chromosomes 3 and 4 (Text S1). OrthoCluster output files listing all detected synteny blocks and their genes are provided in Dataset S2 and Dataset S3. A more detailed discussion of synteny block analysis results can be found in the Supporting Information (Text S1).

To better characterize parasite-specific genes revealed by synteny block analysis, we define three different types of non-syntenic regions (inset Figure 4 and Figure S3). A subtelomeric region (STR) is defined as the genomic region from the most distal gene on a chromosome arm to the first syntenic gene that is part of an imperfect synteny block (there are two such STRs on each chromosome). A synteny breakpoint region (SBR) is defined as a genomic region *between* imperfect synteny blocks. A synteny gap region (SGR) is defined as the genomic region that interrupts perfect synteny *within* imperfect synteny blocks due to the presence of one or more consecutive non-syntenic genes (defined here either as parasite-specific genes that do not have a predicted ortholog in the compared species or as genes that do have a predicted ortholog in the compared species but not syntenic).

**Figure 6:**
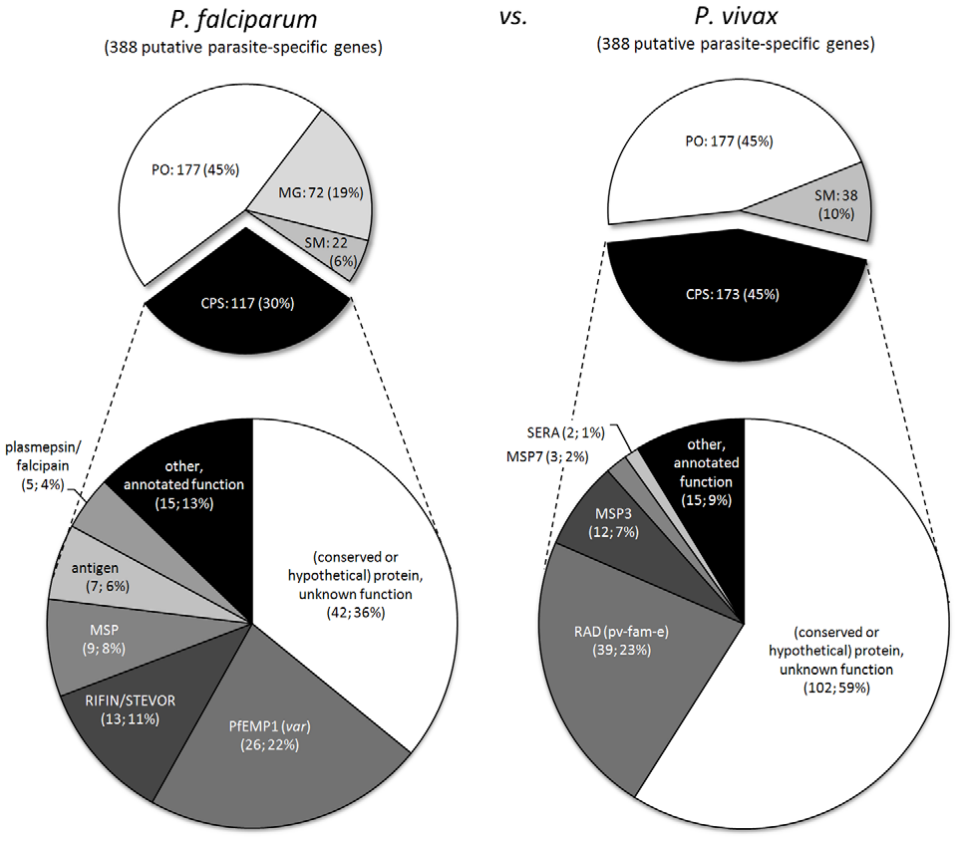
*P. falciparum*-specific genes in chromosome-internal regions enriched with virulence genes. Putative parasite-specific genes identified at SGRs and SBRs between *P. falciparum* (388 genes) and *P. vivax* (388 genes) were examined within their syntenic context (upper two diagrams). Differences considered as non-reliable were excluded from further analysis, including positional orthologs (PO), potential missing genes (MG), and potential split or merged genes (SM). Confirmed parasite-specific (CPS) genes were examined for annotated functions (lower two diagrams). *P. falciparum*-specific genes (lower left diagram) were found to be enriched (FDR-adjusted p-value<0.05) for known virulence factors with associated GO biological processes *pathogenesis* (GO:0009405), *adhesion to host* (GO:0044406), *cell adhesion* (GO:0007155), and *defense response* (GO:0006952), suggesting potential virulence-associated functions for genes currently not implicated in human virulence.

**Figure 7:**
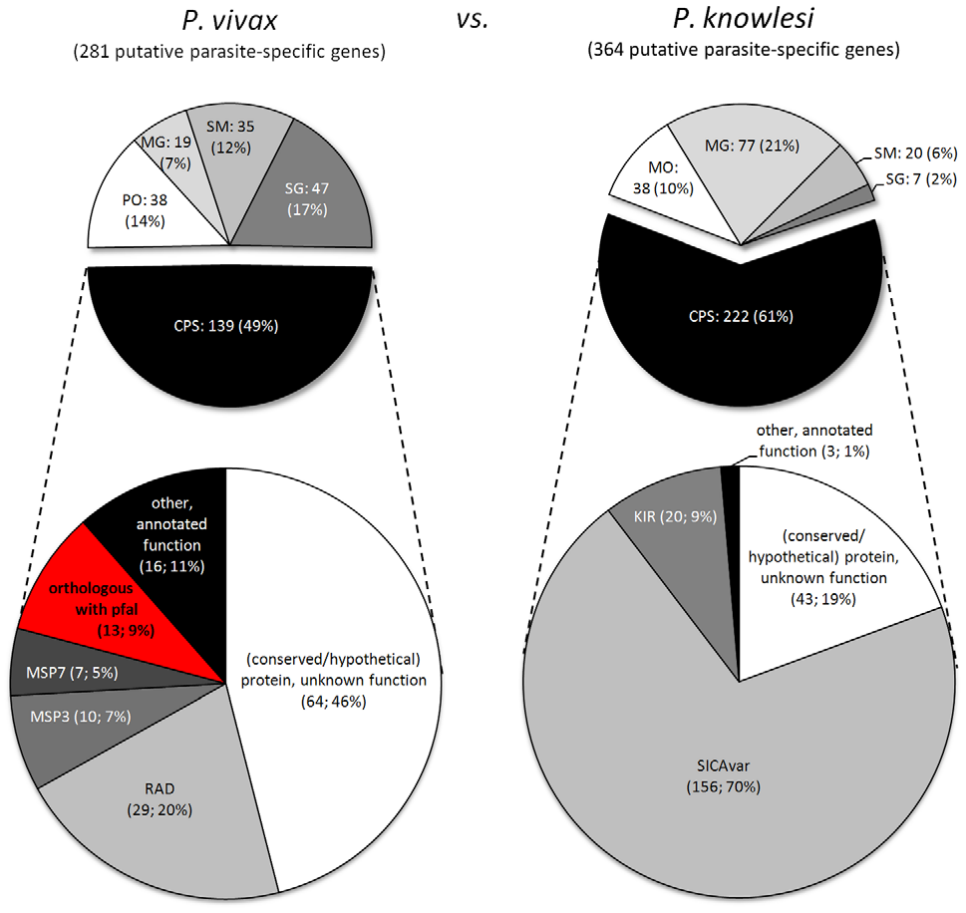
Human parasites *P. falciparum* and *P. vivax* share 13 syntenic orthologs that are absent in the monkey parasite *P. knowlesi*. Putative chromosome-internal parasite-specific genes between the human parasite *P. vivax* (281 genes) and the closely related macaque monkey parasite *P. knowlesi* (364 genes) were examined within their syntenic context. Slices in white and shades of gray in the upper two diagrams show excluded questionable parasite-specific genes, including differences due to positional orthologs (PO), potential missing genes (MG), sequence gaps (SG), and potential split/merged genes (SM). Black slices represent confirmed parasite-specific (CPS) genes examined for their function. Of the 139 confirmed *P. vivax*-specific genes, 13 genes (9%, shown in red and in Table 2) have a syntenic ortholog in *P. falciparum* and thus represent genes present in both human parasites but absent in *P. knowlesi*.

Detected imperfect synteny blocks allowed us to examine chromosome-internal parasite-specific genes within their syntenic contexts (Text S1). In total, syntenic examination confirmed 117 of 388 (30%) *P. falciparum*-specific genes and 173 of 388 (45%) *P. vivax*-specific genes in chromosome-internal regions between *P. falciparum* and *P. vivax* (Figure 6). Between *P. vivax* and *P. knowlesi*, 139 of 281 (49%) *P. vivax*-specific genes and 222 of 364 (61%) *P. knowlesi*-specific genes were confirmed (Figure 7). Thus, depending on the comparison, SGRs and SBRs were found to contain 16–58% of the total number of parasite-specific genes in each species (Text S1 and Table S6), representing a considerable amount of the total parasite-specific gene content in each species. The gene content of these SGRs and SBRs is discussed in the following sections.

### Human parasites share genes absent in *P. knowlesi* that are specifically up-regulated in gametocytes or sporozoites

Although *P. vivax* and *P. knowlesi* are phylogenetically much more closely related to each other than *P. vivax* is to *P. falciparum* (Figure S1 and [33]), we identified 13 genes that are syntenic orthologs between *P. vivax* and *P. falciparum* but absent from syntenic regions in *P. knowlesi* (Figure 7, red slice). Indeed, orthologs of those genes were not found anywhere in the *P. knowlesi* genome, even after screening *P. knowlesi* genomic sequences with genBlastG to account for unannotated genes (see Materials and Methods).

Table 2 shows *P. falciparum* orthologs of these 13 genes together with their degree of conservation in *P. vivax* and gene expression data (see Table S10 for a list of the same genes but including graphical expression profiles). Three genes (PFA0380w, PF14_0236, and PF10_0185) show no or only weak expression in the intraerythrocytic developmental cycle (IDC) and were found to be specifically upregulated in gametocytes or sporozoites [30], which is consistent with the possibility that they may play a role in parasite development within the mosquito host and hence transmission success between humans. One of these three genes (PFA0380w) is annotated as putative *serine/threonine kinase*. NCBI BLASTP search with this gene revealed that it is much closer related to its *P. vivax* ortholog (PVX_081395; E = 9e-66; PID = 54%) than to any *P. falciparum* paralog (best hit PF13_0258 (TKL3); E = 0.008; PID = 48%), suggesting that the presence of this gene is of functional importance.

**Table 2:** Genes shared between *P. falciparum* and *P. vivax* but absent in *P. knowlesi*.

PfGene (PvPID/OG)	Product	RNA-seq. expr. (IDC)	Protein expr.	Additional information
PFI1405c(44/RODE)	unknown function	Min: 15 hMax: 25 h	TZ; iRBCm	C-terminal TM (InterProScan); antigenic variation (PlasmoDraft)
MAL8P1.126(35/ALVE)	serine protease, putative	Min: 10 hMax: 35 h	oocyst SZ	Deg2 chloroplast peptidase (MEROPS); sole member of clan PA/family S1; SP
PF14_0454(23/RODE)	unknown function	Min: 10 hMax: 35 h	SZ;TZ; (el)GC	calponin-like actin-binding; winged-helix DNA binding; defense response (PlasmoDraft)
PF11_0460(19/RODE)	unknown function	Min: 20 hMax: 35 h	GC; MZ; eStages	upregulated in GC
PFL0170w(19/RODE)	transporter, putative	Min: 20 hMax: 35 h	(l)GC; TZ	12 TM; MFS general substrate transporter
PF11_0361-a(55/RODE)	unknown function	Min: 20 hMax: 35 h	-	6 TM; PQ-loop repeat; SP
PF11_0134(52/RODE)	unknown function	Min: 20 hMax: 35 h	-	TM; DUF1704 member (conserved in many species)
MAL13P1.107(34/----)	unknown function	Min: 20 hMax: 35 h	SZ; (l)GC	SP and GPI-anchor; similarity with neighboring rhoptry protein 2 (PF13_0116)
PFL0360c(8/----)	unknown function	Min: 25 hMax: 35 h	-	3 TM; ZF; divergent Pv positional ortholog; similar to serine protease (PlasmoDB)
PF14_0236(12/----)	unknown function	Min: 25 hMax: 35 h	SZ; MZ	ZF; divergent Pv positional ortholog; antigenic variation (PlasmoDraft); upregul. in GC
PFI1216w(52/RODE)	telomeric repeat binding factor 1	Min: -Max: -	-	homeodomain-like; SANT, DNA binding; MYB-like; EST support
PFA0380w(10/RODE)	serine/threonine kinase, putative	Min: -Max: -	SZ	N-terminal TM; divergent Pv positional ortholog; EST support; upregulated in SZ
PF10_0185(28/RODE)	unknown function	Min: -Max: -	-	EST support; upregulated in GC

Shown are all *P. falciparum* genes that were found to have a syntenic ortholog in the second human parasite *P. vivax* but no identifiable ortholog (neither syntenic nor non-syntenic) in the monkey parasite *P. knowlesi*. Four of these genes (MAL8P1.126, MAL13P1.107, PFL0360c, and PF14_0236) also lack an identifiable ortholog in the three rodent parasite genomes and are thus potentially unique to human *Plasmodium* parasites. Genes ordered and grouped by similarity of their IDC expression profile (see legend Table 1). A table with graphical RNA-seq expression profiles for these genes is provided in Table S10. Abbreviations: Pf: *P. falciparum*; Pv: *P. vivax*; PvPID: global protein sequence identity with *P. vivax* ortholog; OG: closest OrthoMCL DB species out-group with predicted ortholog of this gene; ALVE: Alveolates; RODE: rodent malaria parasites; iRBCm: infected red blood cell membrane (PIESPs)/Schizont; SZ: sporozoites; (el)GC: (early/late) gametocytes; TZ: trophozoite; MZ: merozoites; SC: schizont; ZF: zinc finger domain; TM: predicted transmembrane domain; SP: predicted signal peptide.

Four genes in Table 2 (MAL13P1.107, MAL8P1.126, PF14_0236, and PFL0360c) lack orthologs also in rodent malaria parasites and are thus potentially unique to human malaria parasites. Three of these genes show only weak expression in the intraerythrocytic developmental cycle (IDC) and two protein products were detected in sporozoites, again pointing towards a possible role of these genes in parasite development within the mosquito host. MAL13P1.107 shows sequence similarity (BLASTP PID 30, E = 2e-33) with the neighboring gene *rhoptry protein 2* [34], suggesting a function during host cell invasion. MAL8P1.126 is annotated as *Deg2 chloroplast peptidases* and is the sole *P. falciparum* member of clan PA [26]. NCBI BLASTP and TBLASTN searches revealed that MAL8P1.126 is conserved in other *Apicomplexa* species but not non-human malaria parasites, suggesting functional gene loss in non-human malaria parasites. It should be emphasized that a syntenic gene of MAL8P1.126 annotated as *DegP-like serine protease 1 precursor* is present in *P. knowlesi* (PKH_011050) but much shorter (409 aa) with very low sequence similarity to MAL8P1.126 (global PID 8; BLASTP e = 6e-7). Thus PKH_011050 is probably a non-functional pseudogene. The remaining two putative human malaria parasite-specific genes (PF14_0236 and PFL0360c) have no annotated function. Both contain a predicted zinc finger domain and PF14_0236 is predicted to be involved in antigenic variation [35] and PFL0360c shows similarity to a serine protease [36].

We found indications that *P. knowlesi* could lack a functional copy of *telomeric repeat binding factor 1* (TRF1). Running GeneWise with TRF1 of *P. falciparum* (PFI1216w) against the syntenic region in *P. knowlesi* reveals only residual protein sequence similarity (24% global PID), which is well below the degree of conservation found with *P. vivax* (52% PID) and probably indicative of recent gene inactivation in *P. knowlesi*. Both BLASTP and TBLASTN searches using PFI1216w as query against the complete *P. knowlesi* genome revealed the syntenic region of PFI1216w as best hit. Although almost certainly not linked to parasite transmission success, the potential absence of a fully functional copy of TRF1 in *P. knowlesi* is interesting, because it could offer an explanation for the presence of hundreds of variant surface antigens and telomeric repeats in chromosome-internal regions of the *P. knowlesi* genome (see Discussion).

Functions of the remaining genes shared by *P. falciparum* and *P. vivax* but absent in *P. knowlesi* (Table 2) remain largely unknown. Notably, the 13 identified genes are statistically significantly enriched (p = 0.0159) for genes whose expression is induced during the trophozoite stage (20 h post infection) and peaks during the schizont stage (36 h post infection). It remains to be determined if these genes are therefore also functionally related.

### Chromosome-internal *P. falciparum*-specific genes enriched with virulence genes

Looking at parasite-specific genes identified between the highly virulent parasite *P. falciparum* and the less virulent human parasite *P. vivax*, our analysis recovers many known human virulence genes in *P. falciparum* (Figure 6, bottom left). The largest fraction (26 genes, 22%) of the 117 chromosome-internal *P. falciparum*-specific genes is annotated as chromosome-internal members of the *var* gene family, the prime virulence factors of *P. falciparum* [18]. GO term enrichment analysis with Ontologizer [37] reveals that the 117 *P. falciparum*-specific genes are statistically significantly enriched for GO biological processes *pathogenesis* (GO:0009405; FDR-adjusted p-value = 9e-5) and *adhesion to host* (GO:0044406; p = 0.02), mostly because of the presence of these 26 *var* genes (Table S7). The two second largest subgroups of *P. falciparum*-specific genes are also involved in important pathogenic processes, including nine MSPs and 13 members of the rif/stevor gene family. Enriched GO terms associated with these genes include *cell adhesion* (GO:0007155; p = 2e-25) and *defense response* (GO:0006952; p = 3e-6). More generally, we find *P. falciparum*-specific genes enriched for GO subcellular locations *membrane* (GO:0016020; p = 4e-10) and *host intracellular part* (GO:0033646; p = 8e-4), indicating enrichment for proteins functional at the parasite-host interface.

To identify novel candidate genes potentially linked to severe *P. falciparum* malaria, we removed known virulence genes and retained genes that contain features commonly associated with human virulence genes, including PEXEL motifs, signal peptides, or transmembrane domains. In addition, we retained *P. falciparum*-specific genes predicted to have virulence-associated functions based on gene co-expression or protein interaction data with known virulence genes (‘guilt-by-association’ principle) [35], [38].

Among the resulting 15 genes (Table 3 and Table S11) we found two genes with annotated functions, including a putative *apyrase* (PF14_0297) and a putative *sugar transporter* (PFE1455w) (see Discussion). The remaining 13 genes are of unknown function. Four genes have predicted human virulence-associated functions based on gene co-expression or protein interaction data [35], [38], including *evasion of host defense* (PF07_0107), *antigenic variation* (PFA0360c), *biological adhesion* (PF10_0350), and *immune response* (PF10_0044). PlasmoDB annotates another two genes with GO terms *cell adhesion* (PF13_0071) and *immune response* (MAL8P1.97), respectively. Eleven genes carry a predicted signal peptide or transmembrane domain and thus potentially function at the parasite-host interface. One of them (PF07_0107) carries an additional PEXEL motif and is thus a predicted erythrocyte surface or exported protein. Looking at RNA-seq expression data for genes with unknown function, all but two genes (MAL8P1.97 and PF10_0044) have associated expression evidence during the intraerythrocytic developmental cycle (IDC). Two genes (PF07_0107 and PF13_0194) are constitutively expressed at high levels, one gene peaks at the trophozoite stage (PF10_0350), seven genes peak at the late trophozoite/early schizont stage, and one gene (PFF0335c) peaks during schizont development. Two genes (PF10_0357 and PF10_0342) appear maximally expressed during the schizont-ring stage transition and co-localize with the MSP3 gene cluster on *P. falciparum* chromosome 10, suggesting a function in erythrocyte invasion.

**Table 3:** *P. falciparum* genes absent in *P. vivax* with possible role in human virulence.

PfGene (OG)	Product	RNA-seq expr. (IDC)	Protein expr.	Additional information
PF07_0107(----)	exported protein, unknown function	Min: 40 hMax: 25 h	-	2 TM domains; evasion of host defense (PlasmoDraft, 80%); PX
PF13_0194(----)	probable protein, unknown function	Min: 0 hMax: 35 h	TZ	chr13 MSP7-like gene cluster; SP; 1 TM domain; similar to MSP7-like
PF10_0350(----)	probable protein, unknown function	Min: 15 hMax: 25 h	-	chr10 MSP gene cluster; SP and GPI anchor; biological adhesion (PlasmoDraft, 66%)
PF13_0071(----)	probable protein, unknown function	Min: 15 hMax: 30 h	TZ;SC;RU;SZ	cell adhesion, phosphate transport (PlasmoDB)
PF13_0192(----)	conserved, unknown function	Min: 20 hMax: 30 h	TZ;SC;RU;iRBCm	chr13 MSP7-like gene cluster; 2 TM domains; NTP activity (PlasmoDraft)
MAL13P1.106(----)	probable protein, unknown function	Min: 15 hMax: 30 h	(el)GC	SP; 1 TM domain; upregulated in GC
PF14_0708(PLASM)¥	probable protein, unknown function	Min: 20 hMax: 35 h	eGC;MZ	2 TM domains; upregulated in GC; predicted paralog in *P. falciparum* (MAL8P1.95)
PF14_0297(ALVE)	apyrase, putative	Min: 20 hMax: 30 h	-	predicted signal anchor and 2 TM domains; converts ATP to AMP; purine metabolism
PFE1455w(PROT)	sugar transporter, putative	Min: 20 hMax: 35 h	-	12 TM domains; glycoside-pentoside-hexuronide:cation symporter (GPH) family
PFA0360c(----)	conserved, unknown function	Min: 25 hMax: 30 h	-	SP; 1 TM domain; antigenic variation (OPI); upregulated in GC & SZ
PFF0335c(----)	probable protein, unknown function	Min: 25 hMax: 35 h	eGC;TZ;MZ;eSC;RU	membrane protein from schizonts; possible rhoptry/surface protein; SP
PF10_0357(----)	probable protein, unknown function	Min: 20 hMax: 0 h	-	chr10 MSP gene cluster; conserved RESA domain (CCD: PTZ00341)
PF10_0342(----)	probable protein, unknown function	Min: 30 hMax: 40 h	-	chr10 MSP gene cluster; SP
MAL8P1.97(ALVE)	hypothetical protein	Min: -Max: -	oocyst SZ	12 TM domains; immune response, MDR trans-porter domain (PlasmoDB); homolog in *T. parva*
PF10_0044(----)	hypothetical protein	Min: -Max: -	-	WD40 repeat-like; full EST support; immune response (PlasmoDraft, 80%); upregulated in SZ

Selected subset of identified chromosome-internal genes present in the highly virulent human parasite *P. falciparum* but absent in the less virulent human parasite *P. vivax*. Potential virulence-associated function of these genes is predicted based on characteristics shared with known virulence factors, including the presence of a PEXEL motif, signal peptides, and transmembrane domains, or is predicted based on co-expression and protein interaction data [35], [38]. Genes ordered and grouped by similarity of their IDC expression profile (see legend Table 1). A table with graphical RNA-seq expression profiles for these genes is provided in Table S11. Abbreviations: OG: closest OrthoMCL DB species out-group with predicted ortholog of this gene; PLASM: Plasmodium; ALVE: Alveolates; PROT: Proteobacteria; SZ: sporozoites; (el)GC: (early/late) gametocytes; TZ: trophozoite; MZ: merozoites; SC: schizont; iRBCm: infected red blood cell membrane (PIESPs)/Schizont; RU: erythrocyte rupture; OPI: ontology-based pattern identification; TM: predicted transmembrane; SP: predicted signal peptide; PX: PEXEL export motif. ¥ PF14_0708 has a predicted OrthoMCL DB ortholog in *P. vivax* (PVX_123110), but is present as extra copy in *P. falciparum*.

### Uncharacterized gene cluster on *P. vivax* chromosome 6 possibly involved in erythrocyte invasion

**Figure 8:**
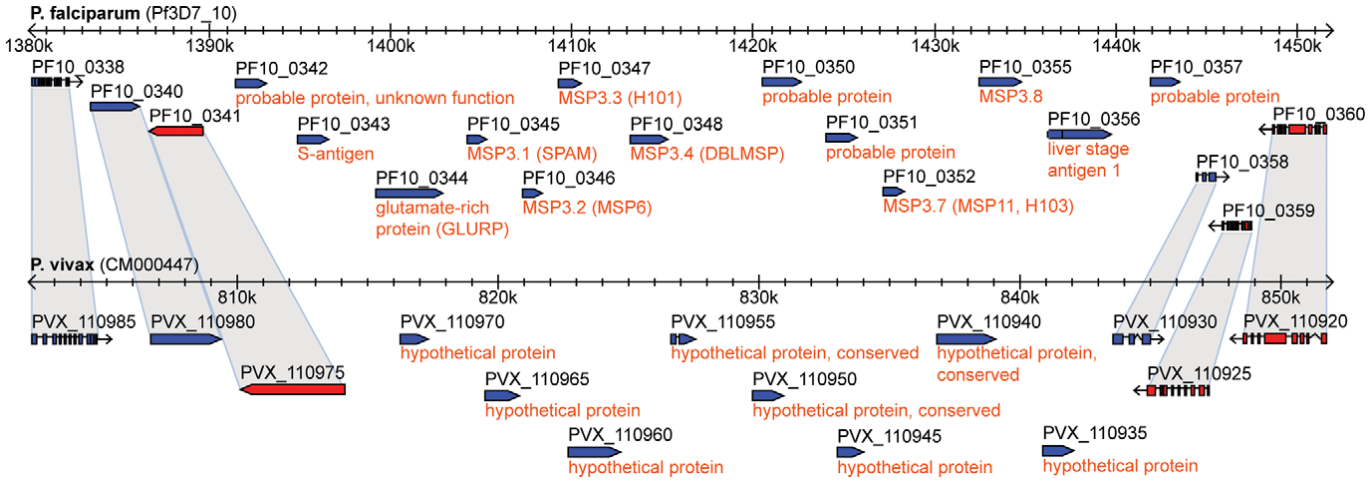
Uncharacterized genes on *P. vivax* chromosome 6 possibly involved in erythrocyte invasion. The upper part of the figure shows a genomic region on *P. falciparum* chromosome 10 with a cluster of 13 *P. falciparum*-specific genes, including the S-antigen, liver stage antigen 1 (LSA1), and five members of the MSP3 gene family, including MSP6. The lower part of the figure shows the syntenic genomic region on *P. vivax* chromosome 6 containing a cluster of eight *P. vivax* hypothetical proteins. Shaded segments indicate orthology. Genes on the forward strand are shown in blue, genes on the reverse strand shown in red. Figure adapted from PlasmoDB 8.0.

Of the 173 identified *P. vivax*-specific genes compared to *P. falciparum* (Figure 6, bottom right), the largest group of genes with named gene products contains members of the previously mentioned RAD gene family (39 genes, 23%), followed by MSP3 genes (12, 7%), and MSP7 genes (3, 2%). Among genes with unannotated function (102 genes, 59%), we identified an interesting and currently uncharacterized *P. vivax* gene cluster of hypothetical proteins likely involved in erythrocyte invasion (Figure 8). This gene cluster is found on *P. vivax* chromosome 6 (position 815,000 to 842,000) and contains eight single-exon genes located on the same strand. The syntenic genomic region in *P. falciparum* maps to the MSP3 gene cluster on chromosome 10 (position 1,390,000 to 1,444,000), which harbors 13 *P. falciparum*-specific single-exon genes also located on the same strand. The *P. falciparum* gene cluster consists of several known antigens and genes involved in erythrocyte invasion, including six members of the MSP3 gene family (including MSP6), the glutamate-rich protein (GLURP) as well as the S-antigen (PF10_0343) and liver stage antigen 1 (PF10_0356). All but one of these genes (PF10_0343) have no predicted ortholog in *P. vivax*. It is possible that the *P. vivax* genes in the syntenic gene cluster on chromosome 6 have a similar function as the *P. falciparum*-specific genes on chromosome 10, which makes them prime candidate genes involved in erythrocyte invasion and interesting targets for further functional characterization. Three other lines of evidence support this conclusion. First, for all but one (PVX_110955) of these eight *P. vivax* genes, top *P. falciparum* BLASTP hits fall into the syntenic *P. falciparum* gene cluster (E-value≤0.05; PID≥28%). Second, all eight *P. vivax* genes carry a predicted signal peptide and are thus likely exported proteins. Third, four *P. vivax* genes (PVX_110945, PVX_088845, PVX_099900, and PVX_089440) peak in expression during the schizont-ring stage transition, which is typical for invasion-related proteins [24].

We identified only few chromosome-internal *P. vivax*-specific genes absent in both *P. falciparum* and *P. knowlesi* that could explain unique biological features of *P. vivax* malaria, in particular the formation of hypnozoites. After excluding questionable open reading frames, only six candidate genes remained (see Materials and Methods). One gene (PVX_099470) has an annotated function and is one of 25 *WD domain*, *G-beta repeat domain containing proteins* in *P. vivax*, all of which occur chromosome-internally. WD-repeat proteins are a large family of proteins found in all eukaryotes and are implicated in a variety of functions, ranging from signal transduction and transcription regulation to cell cycle control and apoptosis. Using PVX_099470 as query, GeneWise predicts a severely truncated syntenic pseudogene in *P. knowlesi* with high identity (56% PID), suggesting recent gene inactivation in *P. knowlesi*. The other five genes have unknown functions. Four genes (PVX_089770, PVX_097730, PVX_110945, and PVX_082710) localize to chromosome-internal RAD, MSP3 (chromosome 10), MSP3 (chromosome 6, putative), and MSP7 gene clusters, respectively, and are thus possibly functionally related to these gene families. The remaining gene (PVX_003710) is a 154 aa single-exon gene with EST expression evidence but of unknown function.

## Discussion

In this study, we compared the genomes of six *Plasmodium* species and proposed several chromosome-internal genes as new candidate genes underlying medically important phenotypic differences, including human pathogenicity, human-mosquito-human transmissibility, and human virulence. Previous studies have shown that important molecular processes at the parasite-host interface, including cytoadherence [13], [18], immune evasion [19], and erythrocyte invasion [20], are typically mediated by species- or species subset-specific genes and that these genes cluster at subtelomeric regions of chromosomes. We hypothesized that human parasites harbor additional human virulence- and pathogenicity genes in chromosome-internal regions. Although we expect parasite virulence and pathogenicity to be primarily the result of gene gain or retention, another possibility not further explored here is that some virulence and pathogenicity is the consequence of adaptive gene loss, as observed in bacteria [39].

We identified 16 genes that are well conserved in the three primate parasites causing human disease but are not found in rodent parasites. Some of these genes could be determinants of primate (and thus human) pathogenicity (Table 1). Most of these 16 genes (9 genes) have predicted OrthoMCL DB orthologs in other Alveolate species (Table 1), suggesting that gene loss in rodent malaria parasites caused these species differences. Multiple lines of evidence suggest that these 16 genes are indeed absent in rodent malaria parasite genomes. First, none of these genes has a predicted Inparanoid or OrthoMCL DB ortholog (neither syntenic nor non-syntenic) in any of the three closely related rodent malaria parasite genomes. Second, screening complete genomic sequences (including the nearly complete chromosome-level assemblies of *P. chabaudi* and *P. berghei* available at PlasmoDB 7.1) with genBlastG did not identify these genes in any of the three rodent malaria parasite genomes. If these genes were present in rodent malaria genomes but mis- or unannotated, then we would expect genBlastG to find them, because all 16 *P. falciparum* genes are well conserved in *P. vivax* and *P. knowlesi*, which have a similar phylogenetic distance to *P. falciparum* as the three rodent parasites. Finally, syntenic genomic regions as determined by flanking orthologs are present in latest chromosome-level assemblies of *P. berghei* and *P. chabaudi* and are assembled without gaps, making it less likely that these genes are absent in rodent parasite genomes due to incomplete genome sequences or assemblies. In some cases as shown in Figure 3, we even find evidence of residual sequence similarity in syntenic regions, which is best explained by (recent) gene loss in rodent malaria parasites. Thus our bioinformatics analysis strongly suggests that these 16 genes are not present in rodent malaria parasite genomes, but ultimate proof will require experimental studies.

Among the 16 putative primate parasite-specific genes were several metabolic enzymes, including three key enzymes of the thiamine (vitamin B1) biosynthesis pathway. This pathway has been proposed as attractive antimalarial drug target because of its absence in human hosts [40], [41]. Our analysis suggests that primate but not rodent malaria parasites synthesize thiamine *de novo* and that rodent malaria parasites depend entirely on thiamine uptake from vertebrate and invertebrate hosts. Indeed, studies have shown that rodent malaria parasites have greatly impaired eryrthrocytic multiplication rates if thiamine is deliberately eliminated from the host [6]. Why particularly primate parasites engage in thiamine biosynthesis is an interesting question. One possibility is that, in primate hosts, thiamine salvage provides the parasite with only insufficient amounts of this essential coenzyme. A more speculative alternative is that thiamine production of the parasite provides the host with this essential enzyme during times when it is only insufficiently available in the host's diet. Interestingly, for all three *P. falciparum* thiamine enzymes, top BLASTP hits outside *Plasmodium* are found in bacteria, including *Clostridium spp.* as top hits in two of three cases (data not shown). In *Clostridium ljungdahlii*, the three enzymes are located next to each other on the same strand and thus form a potential operon (Figure S5), compatible with the possibility that in the common ancestor of *Plasmodium* parasites thiamine biosynthesis was horizontally acquired from bacteria (probably from the mitochondrial or apicoplast genome) and subsequently lost in the common ancestor of rodent malaria parasites. Gene loss in rodent malaria parasites (*vs.* gene gain in primate malaria parasites) as the likely cause for this species-specific difference is supported by residual sequence similarity found in syntenic genomic regions of rodent parasite genomes (Figure 3C).

Comparing the two human parasites *P. falciparum* and *P. vivax* with the monkey parasite *P. knowlesi*, we identified 13 *P. vivax* genes that have a syntenic ortholog in *P. falciparum* but no predicted ortholog (neither syntenic nor non-syntenic) in *P. knowlesi*. The presence of such genes was unexpected because phylogenetically *P. vivax* is much more closely related to *P. knowlesi* than to *P. falciparum* (Figure S1 and [33]). Unlike *P. falciparum* and *P. vivax*, *P. knowlesi* malaria in humans is not endemic in larger parts of the human population and is geographically restricted to forested areas in Malaysian Borneo and peninsular Malaysia [4], [42]. This is most likely due to *P. knowlesi*'s known inability to develop in *Anopheles* species that preferentially feed on humans [6]. We therefore hypothesize that these 13 genes shared by *P. falciparum* and *P. vivax* but absent in *P. knowlesi* may include genes that permit the entry and survival of parasites in anthropophilic human vectors. Consistent with this possibility, three of the 13 genes (PF14_0236, PFA0380w, and PF10_0185) show only weak expression during the IDC and are specifically up-regulated in sporozoites or gametocytes. Notably, four of the identified 13 genes lack orthologs also in rodent malaria parasites and thus could mediate functions specifically required to parasitize humans. The remaining nine genes have predicted orthologs in rodent malaria parasites and thus likely represent cases of gene loss in *P. knowlesi*. Further experimental characterization of these 13 genes is required to confirm a potential role in human transmission success. Eventually, these studies may lead to the development of new transmission blocking strategies or to new ideas how future host switches from monkey to human can be prevented. If the ambitious goal of malaria eradication is to be taken seriously [43], a better understanding of molecular factors contributing to the parasite's ability to complete its life cycle in anthropophilic insect vectors is indispensable.

Comparing the highly virulent human parasite *P. falciparum* with the less virulent human parasite *P. vivax*, we identified 117 chromosome-internal *P. falciparum*-specific genes, many of which have known virulence-associated functions (Figure 6). Subtracting genes with known virulence-associated functions, we identified a subset of 15 genes that we proposed as novel candidate genes potentially linked to severe human malaria (Table 3). Because most of these 15 genes are of unknown function and lack also identifiable orthologs in other species [44], experimental analysis in *P. falciparum* will be required to elucidate their function and to confirm an association with human virulence. The two genes with annotated functions warrant further discussion. The first gene (PF14_0297) is annotated as *apyrase*, which is a membrane-bound enzyme converting ATP to AMP. Apyrases are involved in purine metabolism [26] and, in mosquitoes, are expressed in salivary glands to inhibit blood clotting [45]. The presence of apyrase in *P. falciparum* but not in any other *Plasmodium* parasite (best NCBI BLASTP hit was found in the human apicomplexan parasite *Toxoplasma gondii*) points towards an increased requirement of this enzymatic function in *P. falciparum*. Apyrase has been proposed as possible target for antimicrobial therapies [46], but our finding suggests that its use as antimalarial drug target would be limited to *P. falciparum* malaria. The second gene with annotated function (PFE1455w) is a putative *Na+- or H+-driven sugar symporter* of the GPH family [47] and one of currently six genes annotated with sugar transmembrane transporter activity in *P. falciparum* (GeneDB; GO:0051119). NCBI BLASTP and TBLASTN searches reveal homologs of PFE1455w in *T. gondii* (TGME49_026020) and *Neospora caninum* (NCLIV_046810) but not in any other *Plasmodium* species. Host-derived sugars are an essential nutrient of malaria parasites for intraerythrocytic development [48]. In the absence of gluconeogenesis in malaria parasites [13] additional sugar transporters in the membrane of infected erythrocytes likely allow for more efficient glucose uptake from the blood and thus for faster parasite growth, which can be seen as an adaption towards increased virulence of *P. falciparum*.

Several biological features distinguish *P. vivax* from other sequenced *Plasmodium* species, including preference for reticulocytes and its ability to develop dormant hypnozoite forms in the liver that can cause relapses months or even years after primary infection. We hypothesized that genes present in *P. vivax* but absent in the other sequenced *Plasmodium* species are candidate genes underlying reticulocyte invasion and hypnozoite formation. We identified a currently uncharacterized chromosome-internal gene cluster on *P. vivax* chromosome 6 containing several *P. vivax*-specific genes putatively involved in erythrocyte invasion (Figure 8). Based on synteny, this gene cluster likely encodes for MSPs, including the currently missing *P. vivax* ortholog of *P. falciparum* MSP6 [14]. We expect that further characterization of this gene cluster will result in new insights into *P. vivax*-specific adaptations of erythrocyte invasion. Experimental analyses will be required to test this bioinformatics prediction. In contrast, our search for *P. vivax*-specific genes potentially linked to hypnozoite formation was largely unsuccessful, suggesting that hypnozoite formation has its roots in regulatory differences and is not primarily associated with protein-coding genes that are unique to *P. vivax*.

One peculiarity of the *P. knowlesi* genome is that it has hundreds surface antigens spread all over its genome [15], which we noticed also in our analysis (Figure 7, lower right). How *P. knowlesi* mobilized its once subtelomeric surface antigens and inserted them into chromosome-internal regions is an intriguing question, especially because this must have happened rather recently after the divergence from the common ancestor with *P. vivax* and because transposable elements that could have mediated rapid gene dispersal have not been identified in *Plasmodium* genomes [13]. Our finding that *P. knowlesi* might lack a fully functional copy of TRF1 could provide a possible explanation for this phenomenon. In mammalian cells, telomeric repeat binding factors play a pivotal role in protection and maintenance of telomeres [49]. Partial or complete loss of function of *telomere repeat binding factor 1* in *P. knowlesi* could cause telomere instability, resulting in frequent DNA breakage events near telomeres whose subsequent repair causes broken subtelomeric fragments to be randomly inserted into the *P. knowlesi* genome. Such a mechanism would also explain why *P. knowlesi* harbors telomeric repeat sequences in chromosome-internal regions [15]. Further experimental work can now test this hypothesis and, if confirmed, investigate the important question if the loss-of-function allele of PFI1216w is a fixed wild type allele in the *P. knowlesi* population or a recently introduced mutation, perhaps only present in the sequenced laboratory strain of *P. knowlesi*.

## Materials and Methods

### Genome sequences and gene models

Published chromosome-level assemblies for *P. falciparum*, *P. vivax*, and *P. knowlesi* were downloaded from PlasmoDB version 7.0 [36] (http://plasmodb.org). *P. chabaudi* and *P. berghei* chromosome-level assemblies were available but unpublished. Therefore, older contig-level assemblies available at PlasmoDB (version 5.5) were used for genome-wide comparisons. The *P. yoelii* contig-level assembly was also downloaded from PlasmoDB 5.5. Annotated gene models (GFF3 format) were downloaded from PlasmoDB 7.0 (*P. falciparum*, *P. vivax*, and *P. knowlesi*) and PlasmoDB 5.5 (*P. yoelii*, *P. chabaudi*, and *P. berghei*). If a gene had multiple isoforms, only longest isoforms ( = longest protein sequence) were kept.

### Homology-based gene model improvement of *P. vivax* and *P. knowlesi*

Missing or incorrectly annotated gene models cause overestimates of genetic differences and, important for this study, false specific-specific genes. We therefore repaired the more obvious defects in *P. vivax* and *P. knowlesi* gene model annotations before genome comparisons. Using two homology-based gene predictors, including our own program genBlastG [50] and the widely used and well established program GeneWise [51], an automated pipeline for genome-wide gene model improvement was implemented. Briefly, protein sequences of all protein-coding *P. falciparum* genes (5,317 genes, excluding pseudogenes and shorter isoforms) were used as query to run both genBlastG and GeneWise against *P. vivax* and *P. knowlesi* genomes. To ensure the quality of predicted gene models, only predictions that encoded for protein sequences with high global sequence identity (PID> = 60) with the query gene were kept. If multiple predictions overlapped by more than 5% of their coding exons, only the prediction with the highest PID was kept (filtration step). We use global PID as a measure of sequence conservation because it better captures global similarity between two proteins as compared to for example the BLAST E-value, which measures local sequence similarity and is more prone to various biases, including sequence composition. In a subsequent merging step, predicted and existing gene models were merged into a hybrid gene set, retaining predictions that (a) did not overlap with existing gene models or (b) showed a PID improvement of at least 5% over overlapping existing gene models. As in the filtration step, existing and predicted gene modes were considered as overlapping if more than 5% of their coding exons overlapped. The hybrid gene set served as basis for all subsequent comparisons. A summary of improved gene models, including novel functional annotations predicted with InterProScan [52], can be found in Table S1 (*P. vivax*) and Table S2 (*P. knowlesi*). Novel and improved gene models for both *P. vivax* and *P. knowlesi* are provided in Dataset S1 (GFF3 format).

### Identification of primate parasite-specific genes

Because chromosome-level assemblies for rodent malaria parasites that became available with PlasmoDB 7.0 have not yet been published, we used older, contig-level assemblies (PlasmoDB version 5.5) and a synteny-independent, BLAST-based approach for the initial genome-wide screening for species subset-specific genes. Briefly, complete proteomes of *P. falciparum* (5,317 proteins), *P. vivax* (5,156), *P. knowlesi* (5,143), and *P. yoelii* (7,802) were used as query to run both NCBI BLASTP (version 2.2.21) [53] and genBlastG (version 1.28) [50] against the other five proteomes and genomes, respectively (including *P. berghei* and *P. chabaudi*). Top hits of both BLASTP and genBlastG were used to compute global PIDs with the query protein using ClustalW (version 1.83; BLOSUM62; default parameters) [54]. If the best BLASTP hit was different from the predicted Inparanoid ortholog then the global PID was also computed between query and Inparanoid ortholog. A query protein was considered as conserved in another genome if the maximum of these three PIDs was ≥40 and as absent if the maximum PID was ≤15. In particular, *P. falciparum* genes were considered primate parasite-specific if conserved in *P. vivax* and *P. knowlesi* but not in *P. berghei*, *P. chabaudi*, and *P. yoelii*. The rather conservative margin between high and low PID (25 percent points) was chosen to exclude insignificant PID differences due to fluctuating protein sequence conservation levels or imperfect gene models. Summarized results of this first initial screening are shown in Figure 1. The complete gene list is provided in Table S8. In a second step, *P. falciparum* orthologs of the 30 putative primate parasite-specific genes were inspected using the newer chromosome-level assemblies of *P. chabaudi* and *P. berghei* available at PlasmoDB 7.1. We only kept *P. falciparum* genes for which (a) genBlastG failed to annotate a gene with a minimum global PID of 15 in the entire genome and (b) no gene was present at the expected syntenic region as defined by the position of flanking syntenic orthologs. Sixteen out of the initial 30 genes fulfilled these two criteria and are shown in Table 1.

The two-step process of first screening for putative primate parasite-specific genes against PlasmoDB 5.5 versions of rodent malaria parasite genomes and gene models then verifying the absence of candidate genes in PlasmoDB 7.1 was chosen because, in agreement with pre-publication data use policies, we restricted all genome-scale comparisons to officially published *Plasmodium* genomes. Furthermore, we made no efforts to improve rodent parasite gene models based on the now obsolete PlasmoDB 5.5 genome assemblies, because greatly improved *P. chabaudi* and *P. berghei* gene models became available with PlasmoDB 7.0. It should be emphasized, however, that using the older PlasmoDB 5.5 rodent parasite genome sequences and gene models in the initial screening step did not affect our final results, because all final candidate genes in Table 1 have been verified to be also absent in PlasmoDB 7.1.

### Orthology prediction and synteny block detection

We used OrthoCluster [31] (executable from Dec 17, 2007, downloaded from http://genome.sfu.ca/cgi-bin/orthoclusterdb/download), a program recently developed in our lab, for the gene-based identification of synteny blocks. As input OrthoCluster was provided with genome coordinates of protein-coding genes as well as with gene orthology relationships predicted by Inparanoid (version 4) [55]. Synteny blocks (both perfect and imperfect) were required to have at least two pairs of orthologous genes, irrespective of genomic distance but constrained by the amount of allowed intervening genes. For perfect synteny blocks, we did not allow for any interruptions. For imperfect synteny blocks, we allowed for ≤40% out-map mismatches (*i.e.* genes without predicted orthologs in the other genome) and ≤10% in-map mismatches (*i.e.* genes with, but non-syntenic orthologs in the other genome). These two thresholds were chosen after observing that further increasing the percentages did not result in larger imperfect synteny blocks (Figure S4). OrthoCluster was further run with the *-rs* parameter, which instructs OrthoCluster to report all genes not perfectly preserved in order *and* strandedness as mismatches. This allowed us to localize all genes for which synteny was not perfectly conserved and to examine the nature of those differences in detail. Synteny analysis was performed only on the 14 nuclear chromosomes excluding mitochondrial and apicoplast genomes. Imperfect synteny blocks were visualized using Circos (version 0.52) [56]. Orthology prediction and synteny analysis was performed using our homology-improved gene models.

### Syntenic examination of chromosome-internal parasite-specific genes

To separate questionable differences from likely true genetic differences, we examined all parasite-specific genes in SGRs and SBRs. SGRs were examined in an automated manner using custom Perl scripts. The few SBRs were trickier to deal with due to ambiguous mapping locations in the other genome and were thus examined manually. Briefly, automated BLASTP and GeneWise homology searches were combined with manual visual inspections of non-syntenic regions in a genome browser. Non-random BLASTP sequence similarity (E≤1e-4 and PID≥20) between two ‘syntenic’ parasite-specific genes of which both are flanked by syntenic orthologs was interpreted as evidence of likely orthologous genes missed by Inparanoid. We refer to these genes as positional orthologs. Furthermore, non-random GeneWise alignments (bitscore ≥40) generated in syntenic regions using the putative parasite-specific gene as query are indicative of potentially split/merged genes or missing genes, depending on whether or not alignments overlap with existing gene models. Albeit due to limited sequence similarity GeneWise gene models produced in this step are not entirely reliable, we kept their protein translations for downstream proteomics analyses. In addition, we provide these tentative GeneWise gene models for further inspection (Dataset S4, GFF3 format). Gene structures (gene length, location, number of exons) were also visually examined to validate putative orthologs. We further excluded putative parasite-specific genes for which sequence gaps were present in syntenic regions of the respective other genome, because in this case one cannot reliably exclude the possibility that a syntenic ortholog is present but currently missing from the assembly.

### Identification of human parasite-specific genes

Genes shared by *P. falciparum* and *P. vivax* but absent in *P. knowlesi* (Figure 7 and Table 2) were identified by taking all confirmed *P. vivax*-specific genes absent in *P. knowlesi* (Figure 7) and then excluding all genes without predicted ortholog in *P. falciparum*, considering both Inparanoid orthologs and positional orthologs recovered from Figure 6. In addition, we queried OrthoMCL DB [44] for the presence of orthologs of putative human parasite-specific genes in other species than *P. falciparum* and *P. vivax*. As a last step, we ran genBlastG with remaining *P. falciparum* genes against the entire *P. knowlesi* genome and retained only those candidates that either (a) did not produce a gene model with at least 15% PID and 80% query coverage or (b) produced such a gene model but it overlapped with a *P. knowlesi* gene that had a different known ortholog in *P. falciparum*. The resulting gene list is shown in Table 2. Additional information shown in Table 2 is a compilation of data obtained from searching online databases with *P. falciparum* gene names and sequences, including PlasmoDB 7.1 [36], GeneDB [57], InterPro [52], and NCBI nucleotide and protein archives.

### Identification of *P. falciparum*-specific genes

Starting with all genes in Figure 6 (bottom left diagram) categorized as ‘*other, annotated function*’ (15 genes) and ‘*(conserved or hypothetical) protein, unknown function*’ (42 genes), we excluded genes representing chromosome-internal members of previously described (subtelomeric) gene families (7 genes), genes part of gene families with known members in *P. vivax* (6 genes), genes without expression evidence or questionable open reading frames (9 genes), and genes where visual re-examination revealed the presence of a potential positional ortholog in *P. vivax* that did not meet our similarity threshold for automatic detection (BLASTP E-value<1e-04; PID> = 20). Of the remaining genes we only retained those with potential virulence-associated functions as predicted by (a) the presence of a PEXEL motif, a signal peptide, or a transmembrane domain, or (b) PlasmoDraft [35] or OPI [38]. We further queried OrthoMCL DB [44] for predicted orthologs in other species. One gene (PF14_0708) was found to have a predicted ortholog in *P. vivax*, but was nevertheless retained in the final list because this gene is present as an extra copy in *P. falciparum* (*i.e.* two genes in *P. falciparum* and one in *P. vivax*). As a last step, we ran genBlastG with remaining *P. falciparum* genes against the entire *P. vivax* genome and retained only those candidates that either (a) did not produce a gene model with at least 15% PID and 80% query coverage or (b) produced such a gene model but it overlapped with a *P. vivax* gene that had a different known ortholog in *P. falciparum*. The resulting gene list is shown in Table 3. The complete, unfiltered list of all 117 identified chromosome-internal *P. falciparum* -specific genes is provided in Table S4. As before, the additional information shown in Table 3 is a compilation of data obtained from searching online databases with *P. falciparum* gene names and sequences, including PlasmoDB 7.1, GeneDB, InterPro, and NCBI nucleotide and protein archives. Sequence-independent predictions of virulence-associated functions were obtained from.

### Identification of genes unique to *P. vivax*

To identify genes exclusively present in *P. vivax*, we overlapped the two *P. vivax*-specific gene sets of Figure 6 and Figure 7, which resulted in 81 *P. vivax*-specific genes absent in both *P. falciparum* and *P. knowlesi*. We then excluded genes with named gene products (RAD, MSP7, MSP3, SERA), which resulted in 38 chromosome-internal *P. vivax*-specific genes encoding for hypothetical proteins of unknown function (Table S5). Because median length of encoded protein sequences was short (116 aa), we suspected many false-positive gene predictions among those genes. We therefore further excluded all genes with one or more of the following characteristics: short open reading frames (<100 aa); EST evidence conflicting with the current gene model; coding sequence consisting mostly of low complexity regions or repeat sequences; and the presence of an overlapping gene on opposite strand. Excluding low-confidence ORFs and two other hypothetical genes located near subtelomeric regions resulted in five genes with a likely genuine ORF in chromosome-internal regions.

## Supporting Information

Dataset S1 **Improved** ***P. vivax*** **and** ***P. knowlesi*** **gene models in GFF3 format.**
(TXT)

Dataset S2 **Detected imperfect synteny blocks between** ***P. falciparum*** **and** ***P. vivax*** **.** OrthoCluster ‘.cluster’ output file showing both syntenic and non-syntenic genes of identified imperfect synteny blocks.
(TXT)

Dataset S3 **Detected imperfect synteny blocks between** ***P. vivax*** **and** ***P. knowlesi*** **.** OrthoCluster ‘.cluster’ output file showing both syntenic and non-syntenic genes of identified imperfect synteny blocks.
(TXT)

Dataset S4 **Additional revised** ***P. vivax*** **and** ***P. knowlesi*** **gene models in GFF3 format.** This file contains GeneWise gene models produced during syntenic examination of putative parasite-specific genes using genes from the syntenic genomic region of *P. falciparum* genes as query. Some predictions are based on low sequence similarity (down to ∼15% global protein sequence PID) and should therefore be considered preliminary.
(TXT)

Figure S1 **Phylogeny (A) and selected genome features (B) of the six** ***Plasmodium*** **genomes compared in this study.** The phylogenetic tree was computed with proml/PHYLIP v3.68 based on concatenated protein sequences from 50 randomly chosen well conserved nuclear 1-to-1 orthologs (see Text S1). Out-group species *Babesia bovis* not shown. Branch lengths drawn to scale. Scale bar represents 0.025 amino acid substitutions per site, and numbers at branch points represent bootstrap values from 1,000 iterations. Abbreviations: n/a … not available or not applicable; n/d … not determined; CDS: coding sequence; EST: expressed sequence tag. ¥ Includes mitochondrial and apicoplast genome. †Excluding 447 contigs likely representing DNA contamination from host species *Saimiri boliviensis boliviensis*. ‡Inferred from presence of consensus telomere tandem repeat sequence GGGTT(T/C)A at chromosome ends. § Only longest isoforms.
(DOC)

Figure S2 **Examples of improved gene models in** ***P. vivax*** **and** ***P. knowlesi*** **.** Panel A shows a newly identified 60S ribosomal protein (L39) in *P. knowlesi* (PKH_113715), which has 86% global protein sequence identity (PID) and 98% coverage with its *P. falciparum* ortholog PFF0573c. Panel B shows two *P. vivax* split genes (PVX_090855 and PVX_090860) merged into a single gene (PVX_090860). The revised gene model is supported by EST evidence and, after improvement, recognized as CPW-WPC domain containing protein by InterProScan. Panel C shows a merged gene in *P. knowlesi* (PKH_132400) annotated as dynein-associated protein that was split into two genes (PKH_132400a and PKH_132400b), one of which is subsequently recognized as membrane occupation and recognition nexus (MORN)-motif containing protein. Panel D shows the replacement of a truncated hypothetical protein in *P. vivax* (PVX_088280) with a longer gene model, facilitating its recognition as putative acetyltransferase. Improved gene models shown in yellow. Existing PlasmoDB 7.1 gene models shown in blue (forward strand) or red (reverse strand). The complete set of improved gene models is provided in Dataset S1 (GFF format).
(DOC)

Figure S3 **Different types of non-syntenic regions.** *Subtelomeric regions (STR)* range from the first gene on a chromosome arm to the first syntenic gene (two STRs on each chromosome). *Synteny breakpoint regions (SBR)* are defined as genomic regions *between* imperfect synteny blocks, which may or may not contain genes. *Synteny gap regions (SGR)* are defined as genomic regions that interrupt synteny *within* imperfect synteny blocks due to the presence of one or more parasite-specific genes or non-syntenic orthologs (seven SGRs shown). Shown are two real imperfect synteny blocks identified between *P. falciparum* (chromosome 4) and *P. vivax*. The syntenic architecture depicted here (large STRs, few SBRs, many SGRs) is typical for all investigated *Plasmodium* chromosomes.
(TIF)

Figure S4 **Influence of OrthoCluster** ***-ip*** **and** ***-op*** **parameter on the number of identified imperfect synteny blocks between** ***P. falciparum*** **and** ***P. vivax*** **.** The graph shows the number of identified imperfect synteny blocks between *P. falciparum* and *P. vivax* as a function of allowed in-map mismatches (*-ip* parameter) and out-map mismatches (*-op* parameter). After allowing for 10% in-map mismatches and 40% out-map mismatches the number of identified synteny blocks reaches a plateau and does not decrease further. Similar results were observed between *P. vivax* and *P. knowlesi* (data not shown).
(XLS)

Figure S5 **Gene organization of thiamine biosynthesis genes in** ***Clostridium ljungdahlii*** **suggesting an operonic gene structure.** Protein sequences of the three thiamine biosynthesis genes of *P. falciparum* (PFL1920c, PFE1030c, and PFF0680c) were used as NCBI BLASTP queries to search for homologous proteins (*nr* database). For all three genes top hits outside *Plasmodium* were found in bacteria, with top hits in *Clostridium ljundahlii* in two of three cases. In *Clostridium ljundahlii* the three enzymes are located next to each other and on the same strand, suggesting that they could form an operon in this species. Screenshot adapted from the NCBI Entrez database (http://www.ncbi.nlm.nih.gov/sites/entrez?db=gene&cmd=retrieve&list_uids=9446921).
(DOC)

Table S1 **List of improved gene models in** ***P. vivax*** **.** The list includes new and replaced gene models together with characteristics of query and target genes (length, number of exons), EST support, genomic location, method of prediction, sequence conservation levels, query coverage, and newly predicted domains after improvement.
(XLS)

Table S2 **List of improved gene models in** ***P. knowlesi*** **.** The list includes new and replaced gene models together with characteristics of query and target genes (length, number of exons), genomic location, method of prediction, sequence conservation levels, query coverage, and newly predicted domains after improvement.
(XLS)

Table S3 **Gene orthology and synteny between** ***P. falciparum*** **and** ***P. vivax*** **as well as between** ***P. vivax*** **and** ***P. knowlesi*** **.** Ortholog groups and synteny blocks were identified with Inparanoid4 and OrthoCluster, respectively. Only chromosomal contigs considered for synteny analysis. Orthology prediction included genes from all contigs, chromosomal and non-chromosomal. ‡ Number of protein-coding genes after gene model improvement and excluding annotated pseudogenes and shorter isoforms. § Average percent identity of global protein sequence alignments of all one-to-one orthologs computed with ClustalW. Abbreviations: bp … base pairs; chr … chromosome totals, excluding non-chromosomal contigs.
(DOC)

Table S4 **Complete list of identified parasite-specific genes between** ***P. falciparum*** **and** ***P. vivax*** **.** The list includes both subtelomeric and chromosome-internal parasite-specific genes. Chromosome-internal parasite-specific genes are listed together with non-syntenic regions they fall into (SGR or SBR).
(XLS)

Table S5 **Complete list of identified parasite-specific genes between** ***P. vivax*** **and** ***P. knowlesi*** **.** The list includes both subtelomeric and chromosome-internal parasite-specific genes. Chromosome-internal parasite-specific genes are listed together with non-syntenic regions they fall into (SGR or SBR). Note that numbers of questionable parasite-specific genes in this table are lower than in Figure 7 because questionable parasite-specific identified in the previous comparison with *P. falciparum* were excluded for this table.
(XLS)

Table S6 **Number of parasite-specific genes found in non-syntenic regions before (top) and after (bottom) excluding questionable differences.** Questionable cases of parasite-specific genes and non-syntenic orthologs are detected and removed through a combination of both automatic and manual examination of SGRs and SBRs (see Materials and Methods). ‡ Includes 374 genes (*P. vivax*) and 75 genes (*P. knowlesi*) currently located on non-chromosomal contigs.
(DOC)

Table S7 **Enriched GO terms in** ***P. falciparum*** **-specific genes.** *P. falciparum*-specific genes identified in comparison with *P. vivax* were subjected to GO term enrichment analysis using Ontologizer. GO terms statistically significantly enriched after correcting for multiple testing (P-value<0.05, Benjamini-Hochberg correction) are highlighted in red.
(XLS)

Table S8 **List of species subset-specific genes as identified by BLAST and genBlastG.** Genes in this table underlie the Venn diagram of Figure 1. They are listed together with functional annotations and sequence conservation levels as determined with BLASTP and genBlastG.
(XLS)

Table S9 **Genes conserved in primate parasites but absent in rodent parasites.** Same genes as in Table 1 but including graphical RNA-seq expression profiles.
(PDF)

Table S10 **Genes shared between** ***P. falciparum*** **and** ***P. vivax*** **but absent in** ***P. knowlesi*** **.** Same genes as in Table 2 but including graphical RNA-seq expression profiles.
(PDF)

Table S11 ***P. falciparum*** **genes absent in** ***P. vivax*** **with possible role in human virulence.** Same genes as in Table 3 but including graphical RNA-seq expression profiles.
(PDF)

Text S1 **Supplementary information.** Additional information about *Plasmodium* genome features and species phylogeny, homology-based gene model improvement, synteny block analysis, syntenic examination of chromosome-internal parasite-specific genes, compositional bias of *Plasmodium* genomes, and enriched GO terms of *P. falciparum*-specific genes.
(DOC)
