# Supplementary material for: Genome Comparison of Human and Non-Human Malaria Parasites Reveals Species Subset-Specific Genes Potentially Linked to Human Disease
Source: PLoS Comput Biol. 2011 Dec 22;7(12):e1002320. doi: 10.1371/journal.pcbi.1002320 (PMC3245289; doi:10.1371/journal.pcbi.1002320)
Supplement: Figure S1 — Phylogeny (A) and selected genome features (B) of the six Plasmodium genomes compared in this study. The phylogenetic tree was computed with proml/PHYLIP v3.68 based on concatenated protein sequences from 50 randomly chosen well conserved nuclear 1-to-1 orthologs (see Text S1). Out-group species Babesia bovis not shown. Branch lengths drawn to scale. Scale bar represents 0.025 amino acid substitutions per site, and numbers at branch points represent bootstrap values from 1,000 iterations. Abbreviations: n/a … not available or not applicable; n/d … not determined; CDS: coding sequence; EST: expressed sequence tag. ¥ Includes mitochondrial and apicoplast genome. †Excluding 447 contigs likely representing DNA contamination from host species Saimiri boliviensis boliviensis. ‡Inferred from presence of consensus telomere tandem repeat sequence GGGTT(T/C)A at chromosome ends. § Only longest isoforms. (DOC) [file pcbi.1002320.s005.doc]

**(A)**

**
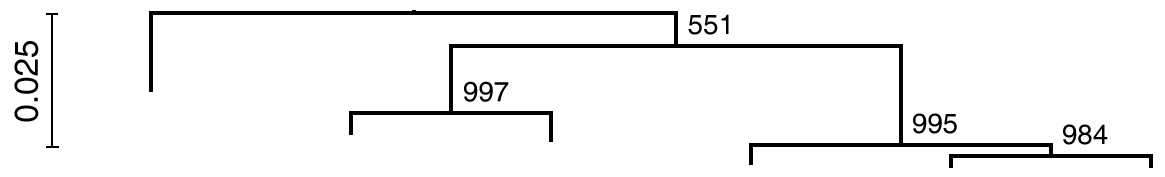
**

**(B)**

|  | ***falciparum***  (human) | ***vivax***  (human) | ***knowlesi***  (monkey) | ***chabaudi*** (rodent) | ***berghei*** (rodent) | ***yoelii*** (rodent) |
| --- | --- | --- | --- | --- | --- | --- |
| **Genome sequence** |  |  |  |  |  |  |
| Year published [reference] | 2002 [13] | 2008 [14] | 2008 [15] | 2005 [16] | 2005 [17] | 2002 [17] |
| Sequence coverage (fold) | 14.5 | 10 | 8 | 4 | 4 | 5 |
| No. nuclear chromosomes | 14 | 14 | 14 | 14 | 14 | 14 |
| PlasmoDB version | 7.1 | 7.1 | 7.1 | 5.5 | 5.5 | 5.5 |
| Sequenced genome size (Mb) | 23.3 | 26.6 † | 23.7 | 16.9 | 18.0 | 20.2 |
| No. contigs | 16 ¥ | 2,301 † | 81 | 10,690 | 7,497 | 2,960 |
| N50 contig size (Kb) | 1,688 | 1,679 | 2,147 | 2.4 | 4.2 | 9.2 |
| G+C content (%) | 19.4 | 45.0 | 38.8 | 24.3 | 23.7 | 22.6 |
| Coding (%, excluding introns) | 53.2 | 48.3 | 47.4 | 51.8 | 50.1 | 50.5 |
| No. gaps (median size Kb) | 160 (0.001) | 17 (3.0) | 219 (1.0) | n/a | n/a | n/a |
| Finished subtelomeres‡ | 27 | 3 | 1 | n/a | n/a | n/a |
| **Protein-coding genes** |  |  |  |  |  |  |
| No. genes §  (pseudogenes) | 5,458  (141) | 5,435  (385) | 5,197  (78) | 15,007 (513) | 12,235 (591) | 7,861 (58) |
| Avg. length of CDS (bp) | 2,283 | 2,164 | 2,178 | 584 | 738 | 1,297 |
| Genes with introns (%) | 54.3 | 52.0 | 51.7 | 33.4 | 40.0 | 54.2 |
| Avg. no. introns per gene | 1.57 | 1.49 | 1.61 | 0.57 | 0.76 | 1.03 |
| EST support (%) | 89 | 54 | n/a | n/a | n/d | n/d |
| Complete EST support (%) | 53 | 10 | n/a | n/a | n/d | n/d |
| Mass spec. evidence (%) | 55 | 0.3 | n/a | n/a | n/d | n/d |
