# Supplementary material for: Genome Comparison of Human and Non-Human Malaria Parasites Reveals Species Subset-Specific Genes Potentially Linked to Human Disease
Source: PLoS Comput Biol. 2011 Dec 22;7(12):e1002320. doi: 10.1371/journal.pcbi.1002320 (PMC3245289; doi:10.1371/journal.pcbi.1002320)

## (A) New protein-coding gene
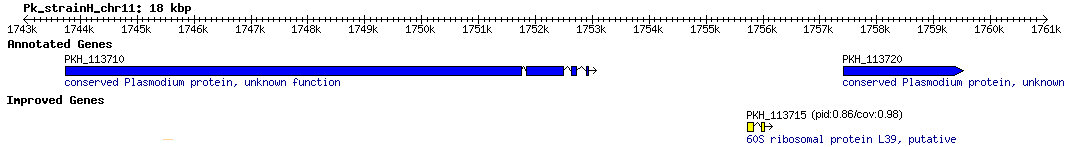


**(B) Split gene that was merged**
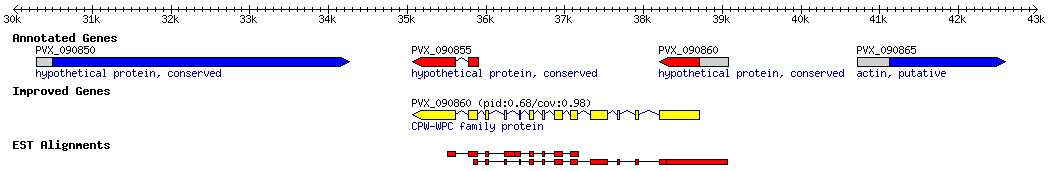


**(C) Merged gene that was split**
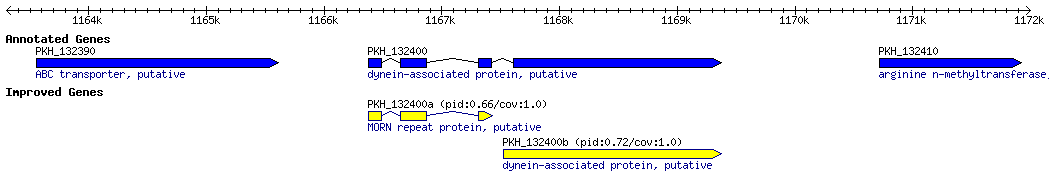


**(D) Gene replacement**
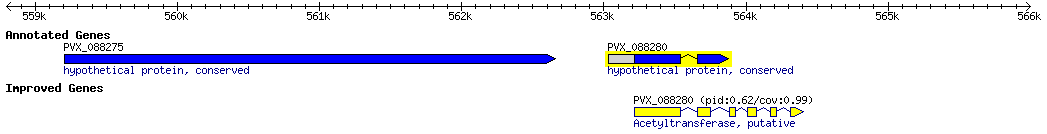

Supplement: Figure S2 — Examples of improved gene models in P. vivax and P. knowlesi . Panel A shows a newly identified 60S ribosomal protein (L39) in P. knowlesi (PKH_113715), which has 86% global protein sequence identity (PID) and 98% coverage with its P. falciparum ortholog PFF0573c. Panel B shows two P. vivax split genes (PVX_090855 and PVX_090860) merged into a single gene (PVX_090860). The revised gene model is supported by EST evidence and, after improvement, recognized as CPW-WPC domain containing protein by InterProScan. Panel C shows a merged gene in P. knowlesi (PKH_132400) annotated as dynein-associated protein that was split into two genes (PKH_132400a and PKH_132400b), one of which is subsequently recognized as membrane occupation and recognition nexus (MORN)-motif containing protein. Panel D shows the replacement of a truncated hypothetical protein in P. vivax (PVX_088280) with a longer gene model, facilitating its recognition as putative acetyltransferase. Improved gene models shown in yellow. Existing PlasmoDB 7.1 gene models shown in blue (forward strand) or red (reverse strand). The complete set of improved gene models is provided in Dataset S1 (GFF format). (DOC) [file pcbi.1002320.s006.doc]
