# Supplementary figures and images for: Genome Comparison of Human and Non-Human Malaria Parasites Reveals Species Subset-Specific Genes Potentially Linked to Human Disease
Source: PLoS Comput Biol. 2011 Dec 22;7(12):e1002320. doi: 10.1371/journal.pcbi.1002320 (PMC3245289; doi:10.1371/journal.pcbi.1002320)

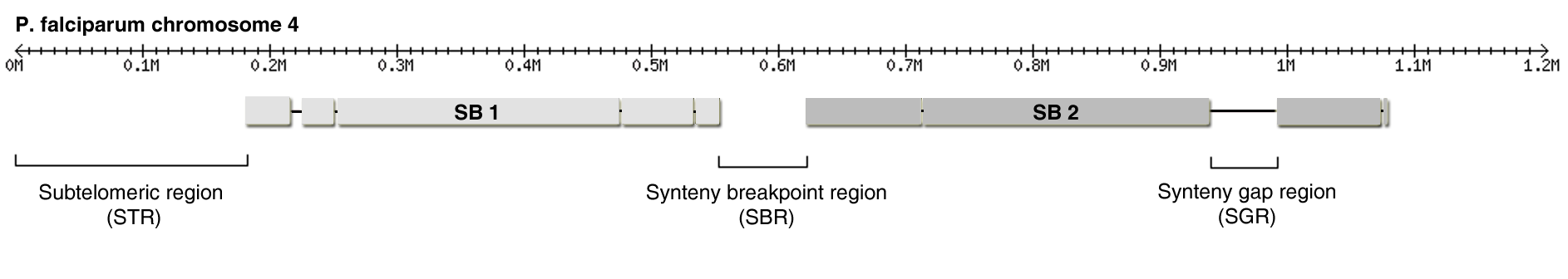

Supplement: Figure S3 — Different types of non-syntenic regions. Subtelomeric regions (STR) range from the first gene on a chromosome arm to the first syntenic gene (two STRs on each chromosome). Synteny breakpoint regions (SBR) are defined as genomic regions between imperfect synteny blocks, which may or may not contain genes. Synteny gap regions (SGR) are defined as genomic regions that interrupt synteny within imperfect synteny blocks due to the presence of one or more parasite-specific genes or non-syntenic orthologs (seven SGRs shown). Shown are two real imperfect synteny blocks identified between P. falciparum (chromosome 4) and P. vivax. The syntenic architecture depicted here (large STRs, few SBRs, many SGRs) is typical for all investigated Plasmodium chromosomes. (TIF) [file pcbi.1002320.s007.tif]

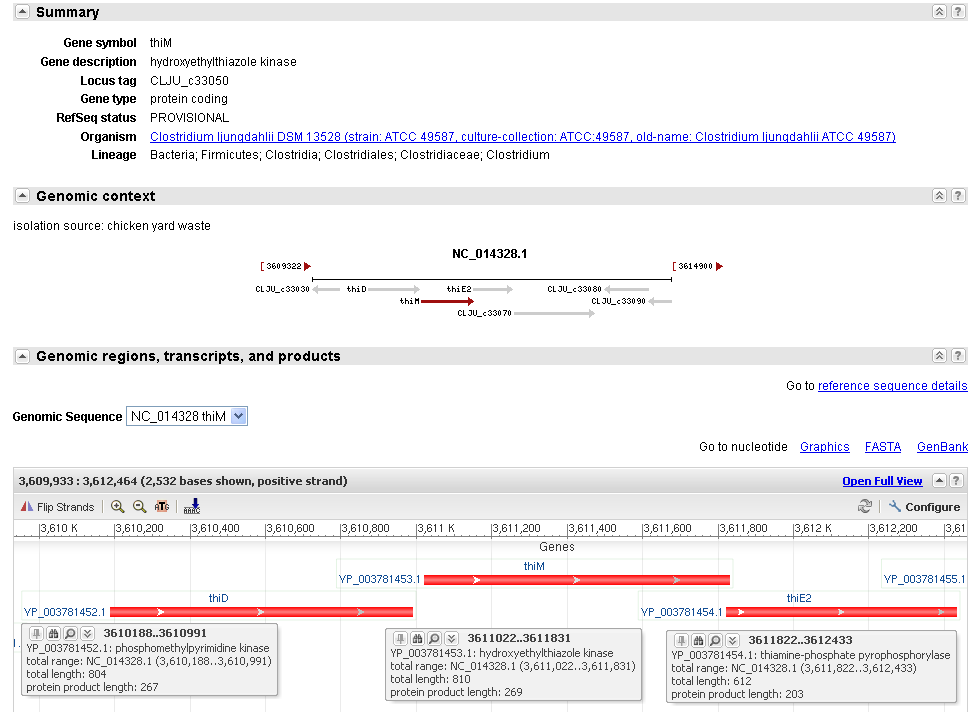


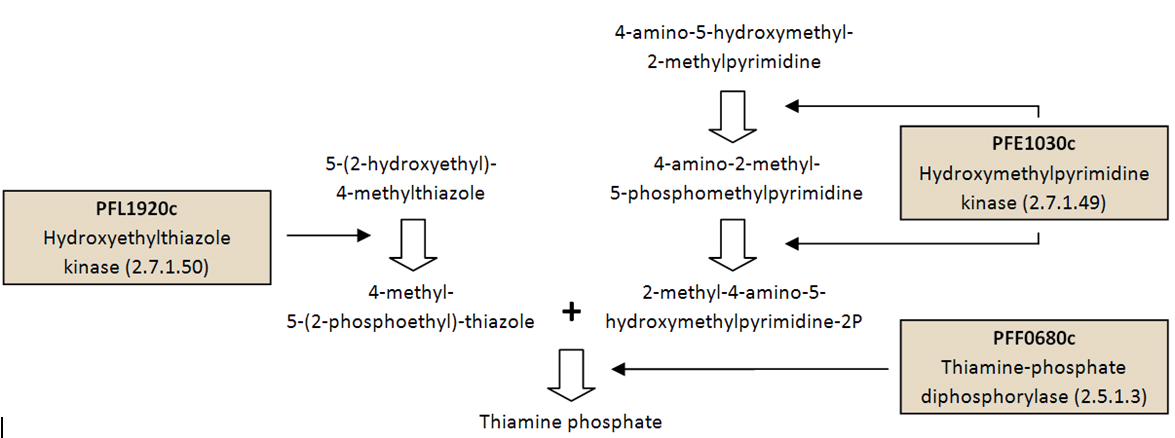

Supplement: Figure S5 — Gene organization of thiamine biosynthesis genes in Clostridium ljungdahlii suggesting an operonic gene structure. Protein sequences of the three thiamine biosynthesis genes of P. falciparum (PFL1920c, PFE1030c, and PFF0680c) were used as NCBI BLASTP queries to search for homologous proteins (nr database). For all three genes top hits outside Plasmodium were found in bacteria, with top hits in Clostridium ljundahlii in two of three cases. In Clostridium ljundahlii the three enzymes are located next to each other and on the same strand, suggesting that they could form an operon in this species. Screenshot adapted from the NCBI Entrez database (http://www.ncbi.nlm.nih.gov/sites/entrez?db=gene&cmd=retrieve&list_uids=9446921). (DOC) [file pcbi.1002320.s009.doc]
