# Supplementary material for: Genome Comparison of Human and Non-Human Malaria Parasites Reveals Species Subset-Specific Genes Potentially Linked to Human Disease
Source: PLoS Comput Biol. 2011 Dec 22;7(12):e1002320. doi: 10.1371/journal.pcbi.1002320 (PMC3245289; doi:10.1371/journal.pcbi.1002320)
Supplement: Table S3 — Gene orthology and synteny between P. falciparum and P. vivax as well as between P. vivax and P. knowlesi . Ortholog groups and synteny blocks were identified with Inparanoid4 and OrthoCluster, respectively. Only chromosomal contigs considered for synteny analysis. Orthology prediction included genes from all contigs, chromosomal and non-chromosomal. ‡ Number of protein-coding genes after gene model improvement and excluding annotated pseudogenes and shorter isoforms. § Average percent identity of global protein sequence alignments of all one-to-one orthologs computed with ClustalW. Abbreviations: bp … base pairs; chr … chromosome totals, excluding non-chromosomal contigs. (DOC) [file pcbi.1002320.s012.doc]

## Table S3: Gene orthology and synteny between *P. falciparum* and *P. vivax* as well as between *P. vivax* and *P. knowlesi*.

|  | ***P. falciparum vs. P. vivax*** | | ***P. vivax vs. P. knowlesi*** | |
| --- | --- | --- | --- | --- |
| **Total no. of genes** ‡ | 5,317 | 5,098 | 5,098 | 5,140 |
| **Genes with ortholog (%)** | 4,459 (83.9%) | 4,478 (87.8%) | 4,726 (92.7%) | 4,767 (92.7%) |
| No. genes one-to-one (%) | 4,373 (82.3%) | 4,373 (85.8%) | 4,610 (90.4%) | 4,610 (89.7%) |
| No. genes one-to-many (%) | 29 (0.6%) | 8 (0.2%) | 20 (0.4%) | 23 (0.4%) |
| No. genes many-to-one (%) | 42 (0.8%) | 80 (1.6%) | 54 (0.1%) | 85 (0.2%) |
| No. genes many-to-many (%) | 15 (0.3%) | 17 (0.3%) | 42 (0.8%) | 49 (1.0%) |
| Avg. PID one-to-one orthologs (%) § | 53.5 | 53.5 | 78.0 | 78.0 |
| **Perfect synteny blocks** | 365 | 365 | 309 | 309 |
| Non-nested synteny blocks | 363 | 363 | 308 | 308 |
| Average size in genes/kb | 12.0/47.0 | 12.0/49.0 | 14.8/61.2 | 14.8/59.8 |
| Median size in genes/kb | 9/33.6 | 9/34.5 | 11/41.1 | 11/40.9 |
| Maximum size in genes/kb | 64/248.5 | 64/267.0 | 70/268.4 | 70/264.8 |
| Genes covered (% chr. total) | 4,343 (81.7%) | 4,352/85.4 | 4,566 (89.6%) | 4,566 (88.8%) |
| Million bp covered (% chr. total) | 17.1 (73.3%) | 17.8 (78.7%) | 18.8 (83.4%) | 18.4 (78.4%) |
| Nested synteny blocks | 2 | 2 | 1 | 1 |
| Average size in genes/kb | 2.5/8.3 | 2/6.7 | 2.0/5.0 | 2/4.2 |
| Median size in genes/kb | 2.5/8.3 | 2/6.7 | 2.0/5.0 | 2/4.2 |
| Maximum size in genes/kb | 3/12.3 | 2/8.4 | 2.0/5.0 | 2/4.2 |
| **Imperfect synteny blocks** | 29 | 29 | 20 | 20 |
| Non-nested synteny blocks | 28 | 28 | 16 | 16 |
| Average size in genes/kb | 170.3/703.2 | 172.1/740.1 | 306.4/1,328.8 | 315.6/1,423.9 |
| Median size in genes/kb | 144.5/563.7 | 152.0/641.1 | 302.0/1,284.7 | 297.5/1,315.9 |
| Maximum size in genes/kb | 433/1,773.4 | 430/1,884.5 | 691.0/2,971.3 | 706/3,089.0 |
| Genes covered (% chr. total) | 4,769 (89.7%) | 4,819 (94.5%) | 4,903 (96.2%) | 5,050 (98.2%) |
| Million bp covered (% chr. total) | 19.7 (84.6%) | 20.7 (91.6%) | 21.3 (94.0%) | 22.8 (97.0%) |
| Species-specific genes | 365 | 399 | 267 | 358 |
| Non-syntenic orthologs | 9 | 10 | 27 | 84 |
| Nested synteny blocks | 1 | 1 | 4 | 4 |
| Average size in genes/kb | 2/4.4 | 2/5.0 | 4/18.2 | 4/17.0 |
| Median size in genes/kb | 2/4.4 | 2/5.0 | 3.5/13.4 | 3.5/12.4 |
| Maximum size in genes/kb | 2/4.4 | 2/5.0 | 7/41.0 | 7/38.9 |

Ortholog groups and synteny blocks were identified with Inparanoid4 and OrthoCluster, respectively. Only chromosomal contigs considered for synteny analysis. Orthology prediction included genes from all contigs, chromosomal and non-chromosomal. ‡ Number of protein-coding genes after gene model improvement and excluding annotated pseudogenes and shorter isoforms. § Average percent identity of global protein sequence alignments of all one-to-one orthologs computed with ClustalW. Abbreviations: bp … base pairs; chr … chromosome totals, excluding non-chromosomal contigs
