# Supplementary material for: Genome Comparison of Human and Non-Human Malaria Parasites Reveals Species Subset-Specific Genes Potentially Linked to Human Disease
Source: PLoS Comput Biol. 2011 Dec 22;7(12):e1002320. doi: 10.1371/journal.pcbi.1002320 (PMC3245289; doi:10.1371/journal.pcbi.1002320)
Supplement: Table S6 — Number of parasite-specific genes found in non-syntenic regions before (top) and after (bottom) excluding questionable differences. Questionable cases of parasite-specific genes and non-syntenic orthologs are detected and removed through a combination of both automatic and manual examination of SGRs and SBRs (see Materials and Methods). ‡ Includes 374 genes (P. vivax) and 75 genes (P. knowlesi) currently located on non-chromosomal contigs. (DOC) [file pcbi.1002320.s015.doc]

**Table S6: Number of parasite-specific genes in non-syntenic regions before (top) and after (bottom) excluding questionable differences.**

|  | ***P. falciparum vs. P. vivax*** | | ***P. vivax vs. P. knowlesi*** | |
| --- | --- | --- | --- | --- |
|  | ***P. falciparum*** | ***P. vivax*** | ***P. vivax*** | ***P. knowlesi*** |
| **Putative parasite-specific genes (% total)** | 1,010 (100%) | 1,073 (100%) | 848 (100%) | 526 (100%) |
| In synteny gap regions (SGR) | 353 (35%) | 386 (36%) | 274 (32%) | 359 (68%) |
| In synteny breakpoint regions (SBR) | 35 (3%) | 1 (0%) | 7 (1%) | 5 (1%) |
| In subtelomeric regions (STR) | 622 (62%) | 686^‡^ (64%) | 567^‡^ (67%) | 162^‡^ (31%) |
| **Excluded questionable differences** | 271 | 215 | 140 | 142 |
| Positional orthologs | 177 | 177 | 38 | 38 |
| Missing genes | 72 | 0 | 20 | 77 |
| Split/merged genes | 22 | 38 | 35 | 20 |
| Sequence gaps | 0 | 0 | 47 | 7 |
| **Confirmed parasite-specific genes (% total)** | 739 (100%) | 859 (100%) | 711 (100%) | 384 (100%) |
| In synteny gap regions (SGR) | 82 (11%) | 172 (20%) | 137 (19%) | 217 (57%) |
| In synteny breakpoint regions (SBR) | 35 (5%) | 1 (0%) | 7 (1%) | 5 (1%) |
| In subtelomeric regions (STR) | 622 (84%) | 686^‡^ (80%) | 567^‡^ (80%) | 162^‡^ (42%) |
