# Supplementary material for: Genome Comparison of Human and Non-Human Malaria Parasites Reveals Species Subset-Specific Genes Potentially Linked to Human Disease
Source: PLoS Comput Biol. 2011 Dec 22;7(12):e1002320. doi: 10.1371/journal.pcbi.1002320 (PMC3245289; doi:10.1371/journal.pcbi.1002320)
Supplement: Table S9 — Genes conserved in primate parasites but absent in rodent parasites. Same genes as in Table 1 but including graphical RNA-seq expression profiles. (PDF) [file pcbi.1002320.s018.pdf]

Table S9: Genes conserved in primate parasites but absent in rodent parasites.

PfGene (PvPID/OG)	Product	RNA-seq expr. (IDC)	Protein expr.	GO biological process	Additional information
MAL13P1.214 (64/VIRI)	phosphoethanolamine N-methyltransferase	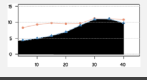	MZ;GC;SC; SZ;RU;TZ	Phosphatidylchol-ine biosynth. pr.	methionine and polyamine metabolism
MAL8P1.202 (53/ALVE)	apicoplast phosphate-idic acid phosphatase	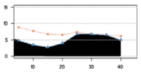	-	-	dolichol metabolism; SP
PF14_0565 (44/----)	unknown function	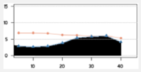	MZ	-	WD40 repeat-like
PF14_0036 (69/ALVE)	acid phosphatase, putative	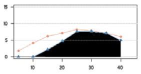	MZ;GC;SC; TZ;RU	-	hydrolase activity
PF11_0186 (65/----)	unknown function	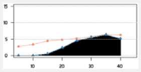	GC;SZ	-	-
PF14_0662 (50/ALVE)	nucleoside transporter, putative	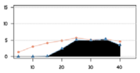	-	-	nucleoside transmem-brane transporter
PFF0680c (40/FIRM)	thiamin-phosphate pyrophosphorylase	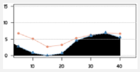	MZ	thiamine bio-synthetic process	-
PFE1030c (67/PROT)	phosphomethylpyrimi dine kinase, putative	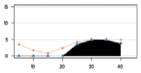	SC;SZ; RU	thiamine bio-synthetic process	-
PFL1920c (76/FIRM)	hydroxyethylthiazole kinase, putative	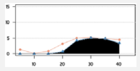	MZ;GC;TZ; SC;RU	thiamine bio-synthetic process	-
MAL7P1.339 (46/ALVE)	Ca++ chelating serine protease, putative	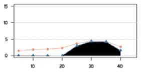	-	-	SCP/Tpx-1/Ag5/PR-1/Sc7 family; SP
PFI0405w (46/----)	unknown function	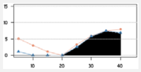		-	1 TM; AP
MAL8P1.111 (42/ALVE)	JmjC domain containing protein	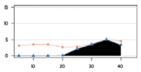	GC	-	[Histone H3]-lysine-36 demethylase
PFL2255w (69/ALVE)	unknown function	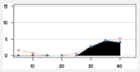	-	ubiquitin cycle	-
PFL1840w (66/ALVE)	unknown function	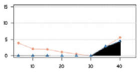	GC;SZ	-	SP; 4 TM; COPI associated
PFL0305c (85/ALVE)	IMP-specific 5'-nucleotidase, putative	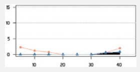	GC; oocyst SZ	nucleotide metabolic process	magnesium ion binding; phosphatase activity
PFI1220w (47/ALVE)	unknown function	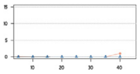	-	-	acyl-CoA N-acyltransfer-ase; upregul. in GC/SZ

Same genes as in Table 1 but including graphical RNA-seq expression profiles taken from PlasmoDB 7.1. RNA-seq expression values represent scaled expression values from the intraerythrocytic developmental cycle (IDC) as reported by Bartfai R *et al.* (2010). Diagram tick marks represent hours post infection along the x-axis (10, 20, 30, and 40 h post infection) and normalized coverage (log2) on the y-axis. For remaining legend please refer to legend of Table 1.
