# Supplementary material for: Genome Comparison of Human and Non-Human Malaria Parasites Reveals Species Subset-Specific Genes Potentially Linked to Human Disease
Source: PLoS Comput Biol. 2011 Dec 22;7(12):e1002320. doi: 10.1371/journal.pcbi.1002320 (PMC3245289; doi:10.1371/journal.pcbi.1002320)
Supplement: Table S10 — Genes shared between P. falciparum and P. vivax but absent in P. knowlesi . Same genes as in Table 2 but including graphical RNA-seq expression profiles. (PDF) [file pcbi.1002320.s019.pdf]

Table S10: Genes shared between *P. falciparum* and *P. vivax* but absent in *P. knowlesi*.

PfGene (PvPID/OG)	Product	RNA-seq. expr. (IDC)	Protein expr.	Additional information
PFI1405c (44/RODE)	unknown function	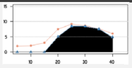	TZ; iRBCm	C-terminal TM (InterProScan); antigenic variation (PlasmoDraft)
MAL8P1.126 (35/ALVE)	serine protease, putative	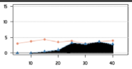	oocyst SZ	Deg2 chloroplast peptidase (MEROPS); sole member of clan PA/family S1; SP
PF14_0454 (23/RODE)	unknown function	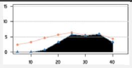	SZ;TZ; (el)GC	calponin-like actin-binding; winged-helix DNA binding; defense response (PlasmoDraft)
PF11_0460 (19/RODE)	unknown function	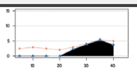	GC; MZ; eStages	upregulated in GC
PFL0170w (19/RODE)	transporter, putative	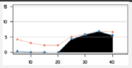	(l)GC; TZ	12 TM; MFS general substrate transporter
PF11_0361-a (55/RODE)	unknown function	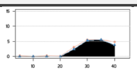	-	6 TM; PQ-loop repeat; SP
PF11_0134 (52/RODE)	unknown function	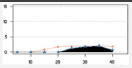	-	TM; DUF1704 member (conserved in many species)
MAL13P1.107 (34/----)	unknown function	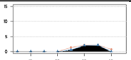	SZ; (l)GC	SP and GPI-anchor; similarity with neighboring rhoptry protein 2 (PF13_0116)
PFL0360c (8/----)	unknown function	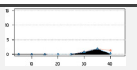	-	3 TM; ZF; divergent Pv positional ortholog; similar to serine protease (PlasmoDB)
PF14_0236 (12/----)	unknown function	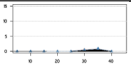	SZ; MZ	ZF; divergent Pv positional ortholog; antigenic variation (PlasmoDraft); upregul. in GC
PFI1216w (52/RODE)	telomeric repeat binding factor 1	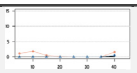	-	homeodomain-like; SANT, DNA binding; MYB-like; EST support
PFA0380w (10/RODE)	serine/threonine kinase, putative	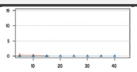	SZ	N-terminal TM; divergent Pv positional ortholog; EST support; upregulated in SZ
PF10_0185 (28/RODE)	unknown function	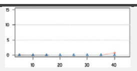	-	EST support; upregulated in GC

Same genes as in Table 2 but including graphical RNA-seq expression profiles taken from PlasmoDB 7.1. RNA-seq expression values represent scaled expression values from the intraerythrocytic developmental cycle (IDC) as reported by Bartfai R *et al.* (2010). Diagram tick marks represent hours post infection along the x-axis (10, 20, 30, and 40 h post infection) and normalized coverage (log2) on the y-axis. For remaining legend please refer to legend of Table 2.
