# Supplementary material for: Genome Comparison of Human and Non-Human Malaria Parasites Reveals Species Subset-Specific Genes Potentially Linked to Human Disease
Source: PLoS Comput Biol. 2011 Dec 22;7(12):e1002320. doi: 10.1371/journal.pcbi.1002320 (PMC3245289; doi:10.1371/journal.pcbi.1002320)
Supplement: Table S11 — P. falciparum genes absent in P. vivax with possible role in human virulence. Same genes as in Table 3 but including graphical RNA-seq expression profiles. (PDF) [file pcbi.1002320.s020.pdf]

Table S11: *P. falciparum* genes absent in *P. vivax* with possible role in human virulence.

PfGene (OG)	Product	RNA-seq expr. (IDC)	Protein expr.	Additional information
PF07_0107 (----)	exported protein, unknown function	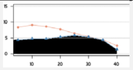	-	2 TM domains; evasion of host defense (PlasmoDraft, 80%); PX
PF13_0194 (----)	probable protein, unknown function	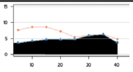	TZ	chr13 MSP7-like gene cluster; SP; 1 TM domain; similar to MSP7-like
PF10_0350 (----)	probable protein, unknown function	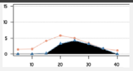	-	chr10 MSP gene cluster; SP and GPI anchor; biological adhesion (PlasmoDraft, 66%)
PF13_0071 (----)	probable protein, unknown function	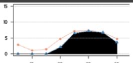	TZ;SC;RU;SZ	cell adhesion, phosphate transport (PlasmoDB)
PF13_0192 (----)	conserved, unknown function	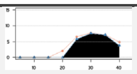	TZ;SC;RU; iRBCm	chr13 MSP7-like gene cluster; 2 TM domains; NTP activity (PlasmoDraft)
MAL13P1.106 (----)	probable protein, unknown function	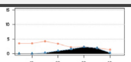	(el)GC	SP; 1 TM domain; upregulated in GC
PF14_0708 (PLASM)¥	probable protein, unknown function	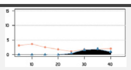	eGC;MZ	2 TM domains; upregulated in GC; predicted paralog in *P. falciparum* (MAL8P1.95)
PF14_0297 (ALVE)	apyrase, putative	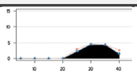	-	predicted signal anchor and 2 TM domains; converts ATP to AMP; purine metabolism
PFE1455w (PROT)	sugar transporter, putative	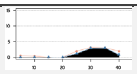	-	12 TM domains; glycoside-pentoside-hexuronide:cation symporter (GPH) family
PFA0360c (----)	conserved, unknown function	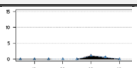	-	SP; 1 TM domain; antigenic variation (OPI); upregulated in GC & SZ
PFF0335c (----)	probable protein, unknown function	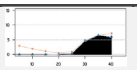	eGC;TZ;MZ; eSC;RU	membrane protein from schizonts; possible rhoptry/surface protein; SP
PF10_0357 (----)	probable protein, unknown function	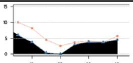	-	chr10 MSP gene cluster; conserved RESA domain (CCD: PTZ00341)
PF10_0342 (----)	probable protein, unknown function	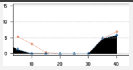	-	chr10 MSP gene cluster; SP
MAL8P1.97 (ALVE)	hypothetical protein	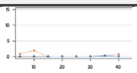	oocyst SZ	12 TM domains; immune response, MDR trans-porter domain (PlasmoDB); homolog in *T. parva*
PF10_0044 (----)	hypothetical protein	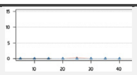	-	WD40 repeat-like; full EST support; immune response (PlasmoDraft, 80%); upregulated in SZ

Same genes as in Table 3 but including graphical RNA-seq expression profiles taken from PlasmoDB 7.1. RNA-seq expression values represent scaled expression values from the intraerythrocytic developmental cycle (IDC) as reported by Bartfai R *et al.* (2010). Diagram tick marks represent hours post infection along the x-axis (10, 20, 30, and 40 h post infection) and normalized coverage (log2) on the y-axis. For remaining legend please refer to legend of Table 3.
