# Supplementary material for: Genome Comparison of Human and Non-Human Malaria Parasites Reveals Species Subset-Specific Genes Potentially Linked to Human Disease
Source: PLoS Comput Biol. 2011 Dec 22;7(12):e1002320. doi: 10.1371/journal.pcbi.1002320 (PMC3245289; doi:10.1371/journal.pcbi.1002320)
Supplement: Text S1 — Supplementary information. Additional information about Plasmodium genome features and species phylogeny, homology-based gene model improvement, synteny block analysis, syntenic examination of chromosome-internal parasite-specific genes, compositional bias of Plasmodium genomes, and enriched GO terms of P. falciparum-specific genes. (DOC) [file pcbi.1002320.s021.doc]

### Features and phylogeny of the six compared *Plasmodium* genomes

The six *Plasmodium* genomes are similar in many aspects (Figure S1). They all consist of 14 nuclear chromosomes and, estimated from genomes sequenced to at least 8x coverage, are similar in size (~25 Mb) and contain a similar number of protein-coding genes (~5,300), about half of which contain at least one intron. For *P. chabaudi*, *P. berghei*, and *P. yoelii*, genome size and number of protein-coding genes are preliminary due to incomplete and fragmented genome sequence data. Despite these similarities, the six genomes show interesting differences as well, in particular in their G+C content. *P. falciparum* and the three rodent parasites have a very low G+C content ranging from 19.4% in *P. falciparum* to 24.3% in *P. chabaudi*. *P. vivax* and *P. knowlesi* have a much more balanced G+C content with 45.0% and 38.8%, respectively. The biological significance of these drastically different G+C contents remains to be appreciated.

Species phylogeny of the six genomes based on 50 randomly chosen conserved nuclear protein-coding genes is in agreement with previous studies [1] and confirms that human parasites do not constitute a monophyletic group (Figure S1). Instead, the human parasite *P. vivax* is more closely related to the monkey parasite *P. knowlesi* than to another human parasite *P. falciparum*. The phylogenetic tree was computed with *proml/PHYLIP* (version 3.68) [2] based on concatenated protein sequences from 50 randomly chosen nuclear 1-to-1 orthologs that had between 60 and 95 PID (ClustalW global protein sequence alignment) with their *B. bovis* ortholog. Concatenated protein sequences were aligned with *ClustalW* (version 1.83; BLOSUM62; default parameters) [3] and alignments were manually trimmed to remove alignment columns with alignment uncertainty, usually found near the start and end of individual protein sequences due to sequence length differences. A bootstrap file with 1000 bootstraps was produced with the *seqboot* program. *proml* was then used with the bootstrap file as input to construct phylogenetic trees (parameters: M=1000; J=Yes; jumble=1) and a consensus tree was computed using the program *consense*. The phylogenetic tree was then displayed using MEGA4 [4].

Quality and completeness of genome assemblies are crucial for characterizing genomic differences. The *P. falciparum* genome sequence is essentially complete with only few unknown bases (‘N’s) remaining in the assembly (Figure S1). *P. vivax* and *P. knowlesi* chromosomal contigs contain larger gaps (= runs of ‘N’s) with median size of 3 kb and 1 kb, respectively. Thus, some genes are expected to be missing from these two assemblies. In rodent parasite genomes, a considerable fraction of genes is likely missing in each genome due to lower read coverage and hence lower assembly quality. We therefore combined the predicted proteomes of the three rodent parasites to increase genetic coverage (see Materials and Methods). As another measure of assembly completeness, we compared the number of fully assembled subtelomeres. Judging from the presence of telomeric repeat sequences at the end of chromosomal contigs, subtelomeres are currently incompletely assembled in all species except *P. falciparum*, which precludes the precise definition of subtelomeric gene content in most species. Interestingly, we failed to identify a canonical telomeric repeat sequence on one arm of P. falciparum chromosome 12, which might thus be incompletely assembled.

We found evidence that *P. vivax* genome sequences are contaminated with DNA from *Saimiri boliviensis boliviens*, which was used as host species in the genome sequencing project. 447 *P. vivax* contigs had NCBI MegaBLAST matches with ≥ 85% sequence identity over ≥ 90% of their length in either the human genome (GRCh37.58) or the genome of *Callithrix jacchus* (version 3.2.1.58). Together, these contigs accounted for 376 kb of the published *P. vivax* genome (PlasmoDB 7.0). It is possible that additional contigs represent host species contamination and all these contigs should be removed from the *P. vivax* genome assembly.

### Homology-based gene model improvement

Accurate definition of genetic differences between *Plasmodium* species requires correctly annotated gene models. Therefore, efforts have been taken in this study to both improve the quality of gene models. Since the initial publication of the *P. falciparum* genome sequence in 2002, large efforts have been taken to experimentally validate and improve *P. falciparum* gene models, including the completion of several cDNA sequencing and proteomics studies (summarized at PlasmoDB at <http://plasmodb.org/plasmo/getDataSource.do?display=detail>). Today, almost ninety percent of *P. falciparum* gene models are supported by expression evidence of some form (Figure S1), more than half of which are fully confirmed by EST evidence. Also, thousands of *P. falciparum* gene models have been critically examined and manually revised in a reannotation effort that started with a weeklong workshop in October 2007, and this effort is still ongoing today with the help of the GeneDB group from WTSI [PlasmoDB, news item from Feb 1, 2008]. Indeed, the first in-depth sequencing-based analysis of the P. falciparum transcriptome using high-throughput sequencing (RNA-Seq) led to the revision of only one out of ten gene models and could not find evidence for new protein-coding genes [5]. Thus, *P. falciparum* protein-coding gene models can be considered of high quality and fairly complete, while this is less certain for the more recently sequenced *Plasmodium* genomes, including *P. vivax* and *P. knowlesi*, where so far much fewer efforts have been taken to improve gene models.

Missing and mispredicted gene models were initially detected in a preliminary whole-genome synteny block analysis between *P. falciparum* and *P.vivax* and between *P. falciparum* and *P. knowlesi*. In this preliminary analysis, we used OrthoCluster to identify imperfect synteny blocks followed by an examination of a few dozen randomly selected parasite-specific genes located at synteny gap regions using GeneWise. In many cases, GeneWise revealed alignments with high sequence similarity in syntenic genomic regions and constructed plausible gene models. From this preliminary analysis we concluded that many putative parasite-specific genes were in fact the result of imperfect gene annotations in *P. vivax* and *P. knowlesi*, caused by merged, split, truncated, and missing gene models. Examples of such defects are shown in Supplementary Figure S2.

Taking advantage of the high-quality gene annotation of *P. falciparum*, we aimed at improving gene models of *P. vivax* and *P. knowlesi* in a genome-wide manner using our recently developed program genBlastG, which is a fast and accurate program for homology-based gene prediction [6] (see Materials and Methods for more details). In total, we identified 53 and 19 new protein-coding genes and revised 165 and 116 existing gene models in *P. vivax* and *P. knowlesi*, respectively. In many cases, revised gene models led to novel functional annotations of known protein motifs and domains, supporting the idea that revisions are correct. In *P. vivax*, 25% (41 of 165) of revised genes produce additional InterProScan [7] hits after revision (Table S1), and in *P. knowlesi* 21% (24 of 116) (Table S2). In addition, multiple lines of evidence suggest that newly predicted genes are indeed missing from current annotations. First, the vast majority (>= 85%; 45/53 genes in *P. vivax* and 16/19 in *P. knowlesi*) of newly identified protein-coding genes produce InterProScan hits, thus sharing similarity with established protein-coding genes. Second, more than half (28 of 53) of newly identified *P. vivax* protein-coding genes have EST expression evidence in PlasmoDB 7.0. No EST data was available for *P. knowlesi* to validate predictions. Third, using the improved gene sets, both number of predicted one-to-one orthologs and perfect synteny block size increased for both *P. vivax* and *P. knowlesi* (see next paragraph). It is also noteworthy that in many cases protein sequences of new or revised gene models revealed the presence of novel signal peptides or transmembrane domains (*P. vivax*: 24 genes; *P. knowlesi*: 18 genes), thus identifying new candidate genes potentially involved in host-pathogen interactions.

### Gene model improvements in *P. falciparum*

During our inspection of putative parasite-specific genes we encountered 10 likely mispredicted *P. falciparum* gene models and one putative novel *P. falciparum* gene, for which we deposited user comments in PlasmoDB. Some fixes were already incorporated into PlasmoDB release 7.0 (personal communication).

### Synteny block analysis

The 28 imperfect synteny blocks identified between *P. falciparum* and *P. vivax* (Figure 4 and Table S3) include all synteny blocks described previously (Figure 1 in [8]) as well as two small (~100 kb) additional imperfect synteny blocks. The first of these two additional synteny blocks contains 21 genes and is an inversion that maps next to the subtelomeric regions on *P. falciparum* chromosome 14 and *P. vivax* chromosome 12. The second synteny block contains 23 protein-coding genes in *P. falciparum* and maps between chromosome-internal regions of *P. falciparum* chromosome 7 and *P. vivax* chromosome 14.

Between *P. vivax* and *P. knowlesi* (Figure 5 and Table S3), the 16 non-nested imperfect synteny blocks span essentially complete chromosomes with two exceptions. One exception maps to *P. knowlesi* chromosome 3, which carries a large inversion of its longer (left) arm relative to *P. vivax* chromosome 3. This large inversion on *P. knowlesi* chromosome 3 was unexpected as *P. vivax* and *P. knowlesi* were previously shown to share perfect chromosomal synteny [8]. It is possible that this inversion is a *P. knowlesi* assembly artifact in PlasmoDB due to presence of flanking sequence gaps in *P. knowlesi* on both sides of the synteny block and absence of this inversion from the *P. knowlesi* genome assembly available at NCBI (accession NC_011904.1). The second exception is a short terminal imperfect synteny block containing 6 genes (28 kb) located on the right arm of *P. vivax* chromosome 4 and mapping to an internal region on *P. knowlesi* chromosome 13. Apart from these two exceptions, OrthoCluster synteny blocks agree with previously published results (Figure 1 in [8]).

Our synteny block analysis confirms previous findings that the genomes of *P. falciparum* and *P. vivax* as well as of *P. vivax* and *P. knowlesi* are highly syntenic except at subtelomeric regions [8,9,10]. OrthoCluster synteny blocks agree well with previous results apart from the above mentioned exceptions. Comparing OrthoCluster results to synteny relationships provided in PlasmoDB 7.1 identified only minor differences (see below). Figure 4 and Figure 5 nicely illustrate that synteny blocks generally span large portions of chromosomes (excluding subtelomeric regions) and frequently map between non-homologous chromosomes, suggesting only few and predominantly large inter-chromosomal rearrangements since divergence from a common ancestor. *P. vivax* and *P. knowlesi* exhibit a particularly striking degree of synteny with almost perfect one-to-one correspondence at the chromosome level (Figure 4 and Table S3), reflecting their closer evolutionary relationship.

Interestingly, small imperfect synteny blocks are extremely rare, suggesting an almost complete absence of small chromosomal rearrangements in *Plasmodium* genomes. We identified only three imperfect synteny blocks with ten or less genes (one of them nested) between *P. falciparum* and *P. vivax* and five (four nested) between *P. vivax* and *P. knowlesi*. This is in stark contrast to other single-cellular eukaryotes like yeast where small rearrangements are prevalent in gene order evolution [11]. Our synteny analysis further reveals that 99.8% of the 3,305 *P. falciparum* genes with predicted orthologs in *P. vivax* have this ortholog positionally conserved (Dataset S2). The cause of this apparent gene order stasis in *Plasmodium* genome evolution remains unknown, but could be a consequence of increased genome stability due to the lack of transposable elements [12]. Alternatively, *Plasmodium* parasites could have problems repairing DNA double-strand breaks because *Plasmodium* species seem to lack a non-homologous end-joining pathway [12]. Without an efficient repair mechanism, double-strand breaks could be strongly negatively selected in haploidic stages of the *Plasmodium* life cycle.

Only minor differences were found between synteny blocks detected by OrthoCluster and synteny blocks that can be obtained from PlasmoDB 7.1. These differences seem to derive from treating SBRs and small genomic rearrangements differently. In PlasmoDB, adjacent synteny blocks extend into the middle of SBRs, whereas OrthoCluster synteny blocks end with the last syntenic gene. At sites involving small genomic rearrangements (12 instances), PlasmoDB splits synteny blocks, whereas OrthoCluster reports one large imperfect synteny block plus smaller (nested) synteny blocks if rearrangements involves at least two adjacent orthologs. Such split synteny blocks in PlasmoDB map to: MAL11:1010000..1059999; MAL14:1250000..1299999; MAL14:1106000..1133999; MAL11:1010000..1059999; MAL8:660000..670999; MAL9:1375000..1433999 (= pfal single-gene inversion of PFI1730w); MAL8:750000..839999 (nested synteny block); MAL12:1570000..1679999 (nested synteny block); MAL12:1150000..1229999 (nested synteny block); MAL14:2570000..2609999 (local rearrangement); MAL6:474000..527999 (local rearrangement in large pkno SGR); MAL12:1420000..1499999 (~100kb SGR in pkno). In one case locating to MAL1:391000..401999 the split of a synteny block in PlasmoDB remains unclear, because after *P. vivax* gene model improvement this region appears to be perfectly syntenic.

### Impact of gene model improvement on ortholog number and perfect synteny block size

As expected, both number of orthologs and perfect synteny block size increased as a result of gene model improvement. Comparing *P. falciparum* with *P. vivax*, the number of 1-to-1 orthologs increased from 4,316 to 4,373 (+1.3%) and the average *P. falciparum* non-nested perfect synteny block size increased from 10.7 genes or 42.0 kb (before improvement) to 12.0 genes or 47.0 kb (after improvement). Similarly, the largest perfect synteny block increased in size from 50 genes (201.2 kb) to 64 genes (248.5 kb). Comparing *P. falciparum* with *P. knowlesi*, the number of 1-to-1 orthologs increased from 4,399 to 4,428 (+0.7%) and the average *P. falciparum* non-nested perfect synteny block size increased from 11.9 genes or 46.8 kb (before improvement) to 12.6 genes or 49.4 kb (after improvement). No increase of the largest perfect synteny block was observed in *P. knowlesi*.

### Syntenic examination of chromosome-internal parasite-specific genes

Syntenic examination of putative parasite-specific genes allowed us to exclude questionable parasite-specific genes and to focus on likely true gene differences between parasites. The majority of putative parasite-specific genes were found to be questionable due to various types of problems. For example, syntenic examination allowed us to reduce the number of putative parasite-specific genes by 70% between *P. falciparum* and *P. vivax* (Figure 6) and 49% between *P. vivax* and *P. knowlesi* (Figure 7), thus dramatically reducing the amount of parasite-specific differences. The largest fraction of excluded differences corresponded to genes with conserved location, direction, syntenic context, and low sequence similarity, which we considered as positional orthologs. It should be mentioned that although we excluded positional orthologs in this analysis because we did not consider them as strictly parasite-specific, we think that divergent positional orthologs are themselves interesting genes for follow-up analysis. Positional orthologs are rapidly evolving and could thus be linked to interesting pathogenic processes at the parasite-host interface, including host immune evasion. Note that numbers of questionable differences in Figure 6 and Figure 7 represent parasite-specific genes and not potential annotation errors. For example, a single split gene can cause up to three false parasite-specific genes considering both genomes. The vast majority (70% to 99%, depending on comparison) of these confirmed chromosome-internal parasite-specific genes was found in small SGRs and only few in SBRs (Table S6). SGRs spread evenly across genomes without obvious bias towards particular chromosomes or chromosomal domains.

After accounting for potential annotation artefacts, our analysis showed that parasite-specific genes remain abundant in chromosome-internal regions and, in fact, represent a substantial fraction of the total parasite-specific gene content in each species: depending on the comparison, SGRs and SBRs harbour 16-58% of the total number of parasite-specific genes (Table S6). This is consistent with earlier finding that *P. falciparum* in comparison with rodent malaria parasites carries many parasite-specific genes in chromosome-internal regions [10] and contrasts with a view that chromosome-internal regions of *Plasmodium* genomes harbour only ‘house-keeping’ genes with few parasite-specific differences.

### Other GO terms enriched in *P. falciparum*-specific genes

GO terms *peptidase activity* (GO:0008233; p=0.009) and *hemoglobin catabolic process* (GO:0042540; p=0.05) were also significantly enriched among chromosome-internal *P. falciparum*-specific genes. Closer inspection revealed three *P. falciparum*-specific aspartic proteases (plasmpesin I, II, and II) and two *P. falciparum*-specific cysteine proteases (falcipain 2a and 2b) associated with these terms. Differential functions of the ten known plasmepsins and four known falcipains have not been completely resolved and thus an assessment of the functional significance of this difference between *P. falciparum* and *P. vivax* is difficult. However, it is tempting to speculate that the maintenance of additional proteases in *P. falciparum* allows for faster growth in erythrocytes due to more efficient haemoglobin digestion, probably an evolutionary adaptation of *P. falciparum* towards increased virulence in humans. *P. falciparum* proteases are known key players in erythrocyte invasion, hemoglobin hydrolysis, and erythrocyte rupture and are also promising and actively studied drug targets [13].

### Compositional bias of *Plasmodium* genomes

Some *Plasmodium* genomes, including *P. falciparum* and the three rodent malaria parasite genomes, are exceptionally AT rich, with AT contents approaching 80% (Figure S1). This compositional bias represents a potential problem for genome and protein sequence analyses, because it can distort sequence database search statistics and scoring.

To handle this technical difficulty, we used BLAST version (v2.2.21) throughout this analysis, which corrects for compositional bias by default (-C2 parameter). genBlastG searches were also performed using BLAST v2.2.21 with the -C2 option turned on. Furthermore, for genBlastG-based gene model improvement and gene conservation analysis, we did not rely on reported BLAST E-values but on percent identity (PID) values of full-length protein sequences computed with ClustalW [3] as a measure of sequence conservation. We used rather stringent PID cutoff values (60% for improving gene models and 40% for looking for conserved genes in primate parasites), expecting that this strategy is less sensitive to compositional bias than using E-values.

For Inparanoid ortholog predictions we also used BLAST v.2.2.21 with the -C2 parameter turned on. Interestingly, we observed that compared to older BLAST versions that did not account for compositional bias, this dramatically reduced the number of reported HSPs within the *P. falciparum* genome and resulted in a few dozen orthologous pairs less between *P. falciparum* and the other *Plasmodium* genomes (data not shown). In other words, the adjustment for compositional bias resulted in a somewhat reduced sensitivity of Inparanoid. However, we were able to recover these orthologs as positional orthologs through our subsequent synteny analysis (Figure 6 and 7) and excluded them from our list of putative parasite-specific genes.

### Bibliography

1. Martinsen ES, Perkins SL, Schall JJ (2008) A three-genome phylogeny of malaria parasites (Plasmodium and closely related genera): evolution of life-history traits and host switches. Mol Phylogenet Evol 47: 261-273.

2. Felsenstein J (1989) PHYLIP -- Phylogeny Inference Package (Version 3.2). Cladistics 5: 164-166.

3. Thompson JD, Gibson TJ, Higgins DG (2002) Multiple sequence alignment using ClustalW and ClustalX. Curr Protoc Bioinformatics Chapter 2: Unit 2 3.

4. Tamura K, Dudley J, Nei M, Kumar S (2007) MEGA4: Molecular Evolutionary Genetics Analysis (MEGA) software version 4.0. Mol Biol Evol 24: 1596-1599.

5. Otto TD, Wilinski D, Assefa S, Keane TM, Sarry LR, et al. (2010) New insights into the blood-stage transcriptome of Plasmodium falciparum using RNA-Seq. Mol Microbiol 76: 12-24.

6. She R, Shih-Chieh Chu J, Uyar B, Wang J, Wang K, et al. (2011) genBlastG: extending BLAST to be a high performance gene finder. Bioinformatics.

7. Mulder N, Apweiler R (2007) InterPro and InterProScan: tools for protein sequence classification and comparison. Methods Mol Biol 396: 59-70.

8. Carlton JM, Adams JH, Silva JC, Bidwell SL, Lorenzi H, et al. (2008) Comparative genomics of the neglected human malaria parasite Plasmodium vivax. Nature 455: 757-763.

9. Carlton JM, Angiuoli SV, Suh BB, Kooij TW, Pertea M, et al. (2002) Genome sequence and comparative analysis of the model rodent malaria parasite Plasmodium yoelii yoelii. Nature 419: 512-519.

10. Kooij TW, Carlton JM, Bidwell SL, Hall N, Ramesar J, et al. (2005) A Plasmodium whole-genome synteny map: indels and synteny breakpoints as foci for species-specific genes. PLoS Pathog 1: e44.

11. Seoighe C, Federspiel N, Jones T, Hansen N, Bivolarovic V, et al. (2000) Prevalence of small inversions in yeast gene order evolution. Proc Natl Acad Sci U S A 97: 14433-14437.

12. Gardner MJ, Hall N, Fung E, White O, Berriman M, et al. (2002) Genome sequence of the human malaria parasite Plasmodium falciparum. Nature 419: 498-511.

13. Rosenthal PJ (2004) Cysteine proteases of malaria parasites. Int J Parasitol 34: 1489-1499.
